# Supplementary material for: Correction of the molecular phenotype of X-linked Dystonia-Parkinsonism reveals a non-canonical function of BRD4
Source: Nat Commun. 2026 May 5;17:4062. doi: 10.1038/s41467-026-72319-6 (PMC13144358; doi:10.1038/s41467-026-72319-6)
Supplement: Supplementary file 1 — Supplementary Information [file 41467_2026_72319_MOESM1_ESM.pdf]

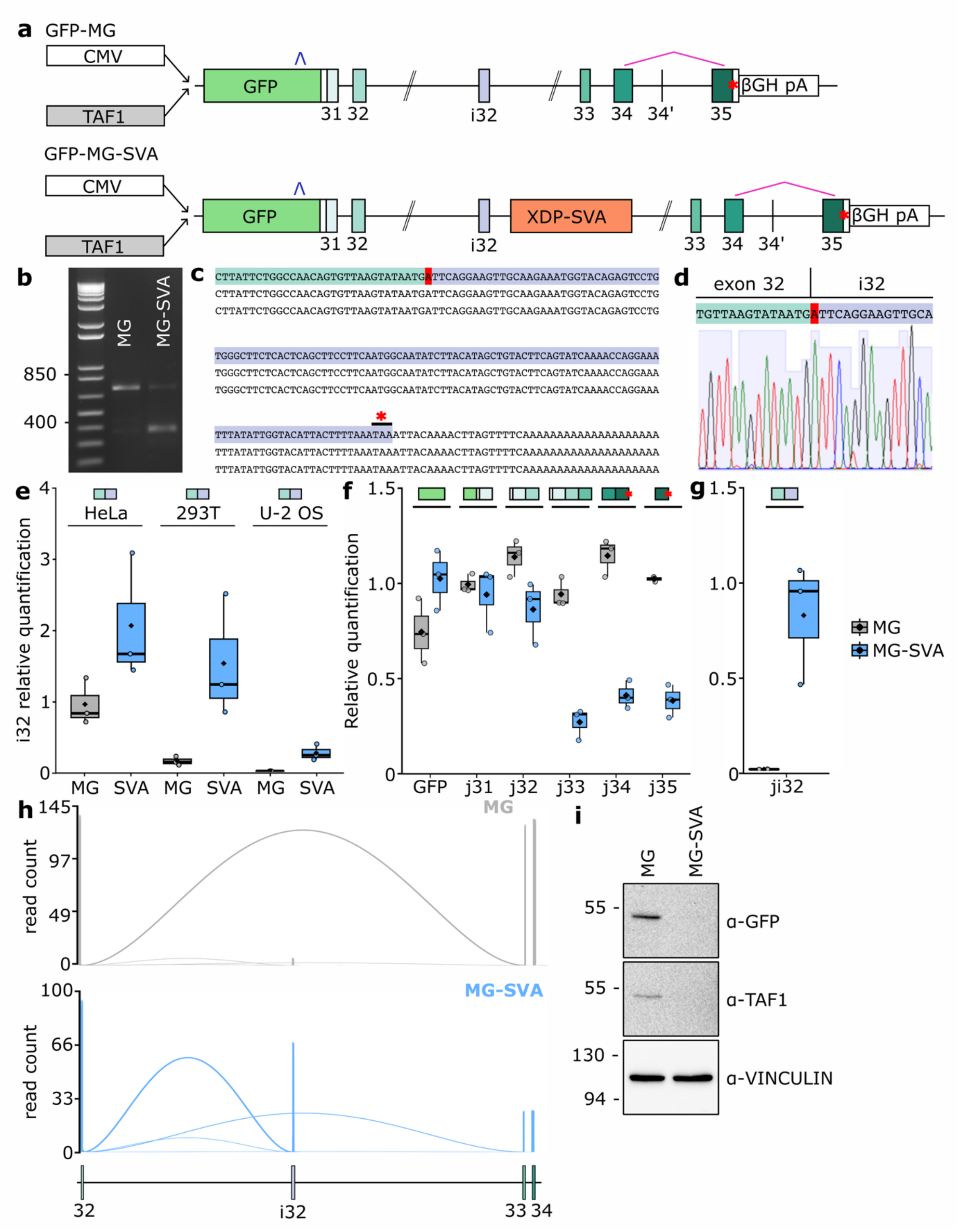

Supplementary Fig. 1: The XDP-SVA *TAF1* reporter recapitulates disease-specific transcript misprocessing across multiple cell lines and promoters.

(a) Schematic representation of a single-color GFP version of the MG and MG-SVA minigene reporters. Exons are shown as boxes and numbered; the XDP-SVA insertion is indicated. The epitope recognized by the TAF1 antibody is indicated in pink; the GFP antibody is shown for reference in blue. The reporter was generated with either a doxycycline-inducible cytomegalovirus (CMV) or the *TAF1* promoter. CMV-driven constructs have been used for

experiments in panels b to e, while panels f to i represent data obtained with the construct under the *TAF1* promoter. (b) 3′ RACE products from MG (full-length products, ~800 bp) and MG-SVA (terminating products, ~400 bp) reporter lines. (c-d) Multiple Sanger sequencing alignment of the predominant 3' RACE product generated by the MG-SVA reporter confirms splicing of exon 32 to the intronic exon i32. (e) Quantification of i32 inclusion in different cell lines (HeLa, 293T and U-2 OS) expressing either the MG or the MG-SVA *TAF1* reporter. (f-g) Relative abundance of splicing junctions for canonical exons (f) and i32 (g) quantified by RT-qPCR for MG and MG-SVA driven by the *TAF1* promoter element. Box plots in (e) to (g) are generated with n=3 biological replicates. (h) Sashimi plot depicting splicing acceptors usage from exon 32 splicing donor site. Read count is depicted as read counts/1000. (i) Immunoblot of TAF1 minigene-derived protein products using GFP and TAF1-specific antibodies.

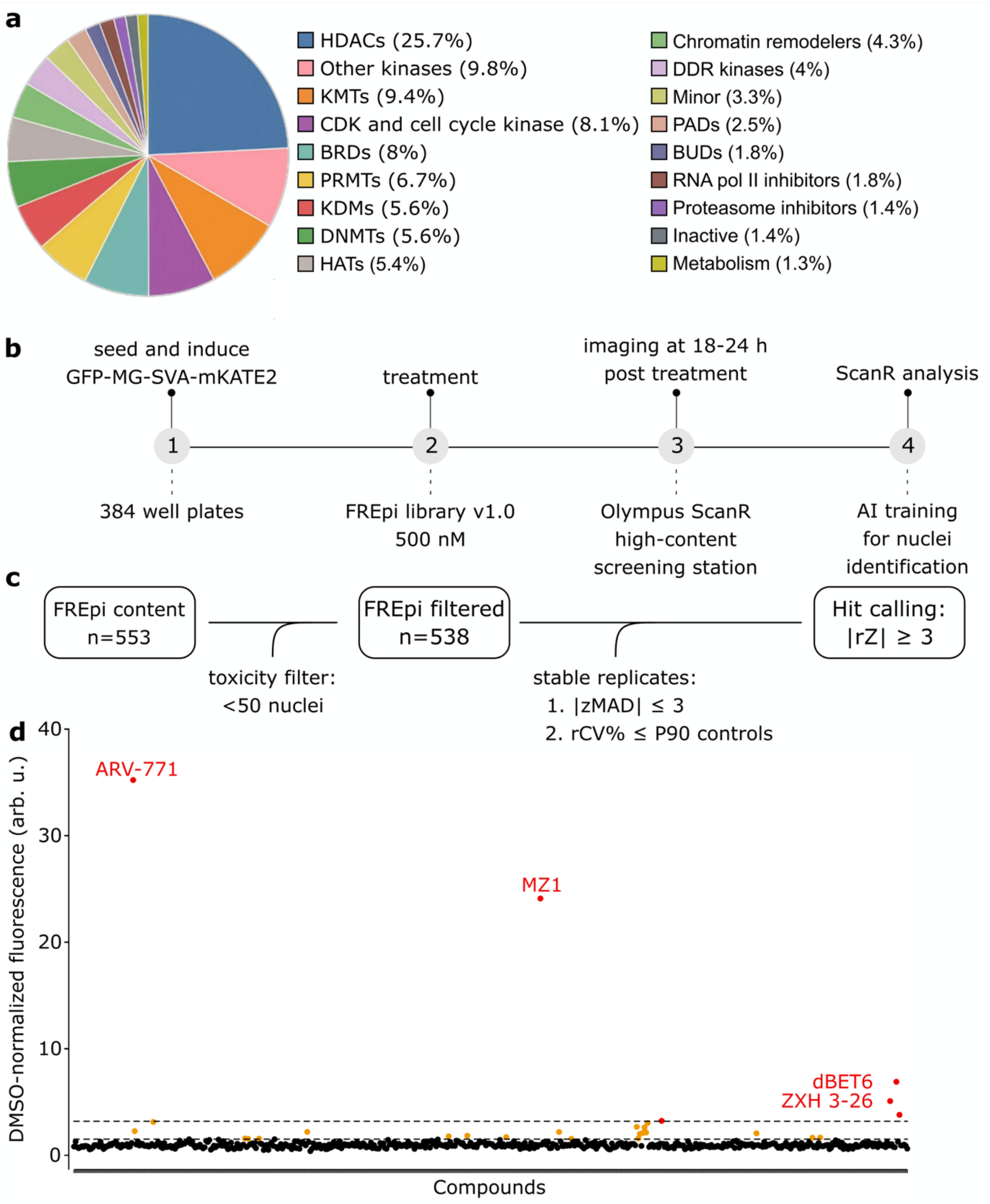

Supplementary Fig. 2: FREpi screening reveals modulation of XDP molecular signature by BET depletion.

(a) Percentage composition for the FREpi key functional small molecule classes. Details reported in Supplementary Table 1. (b) Schematic overview of the high-content screening workflow using the dual-color XDP-SVA *TAF1* reporter line. (c) Post-processing data analysis pipeline showing quality control, filtering, and hit selection steps. zMAD: median absolute deviation-based Z-score; rCV%: robust coefficient of variation; P90: 90th percentile of controls; rZ: robust Z-score. (d) Manhattan plot summarizing the DMSO-normalized mKATE2

fluorescence response (in arbitrary units, arb.u.). The top 1% of hits (red) and the top 2-5% (orange) are shown.

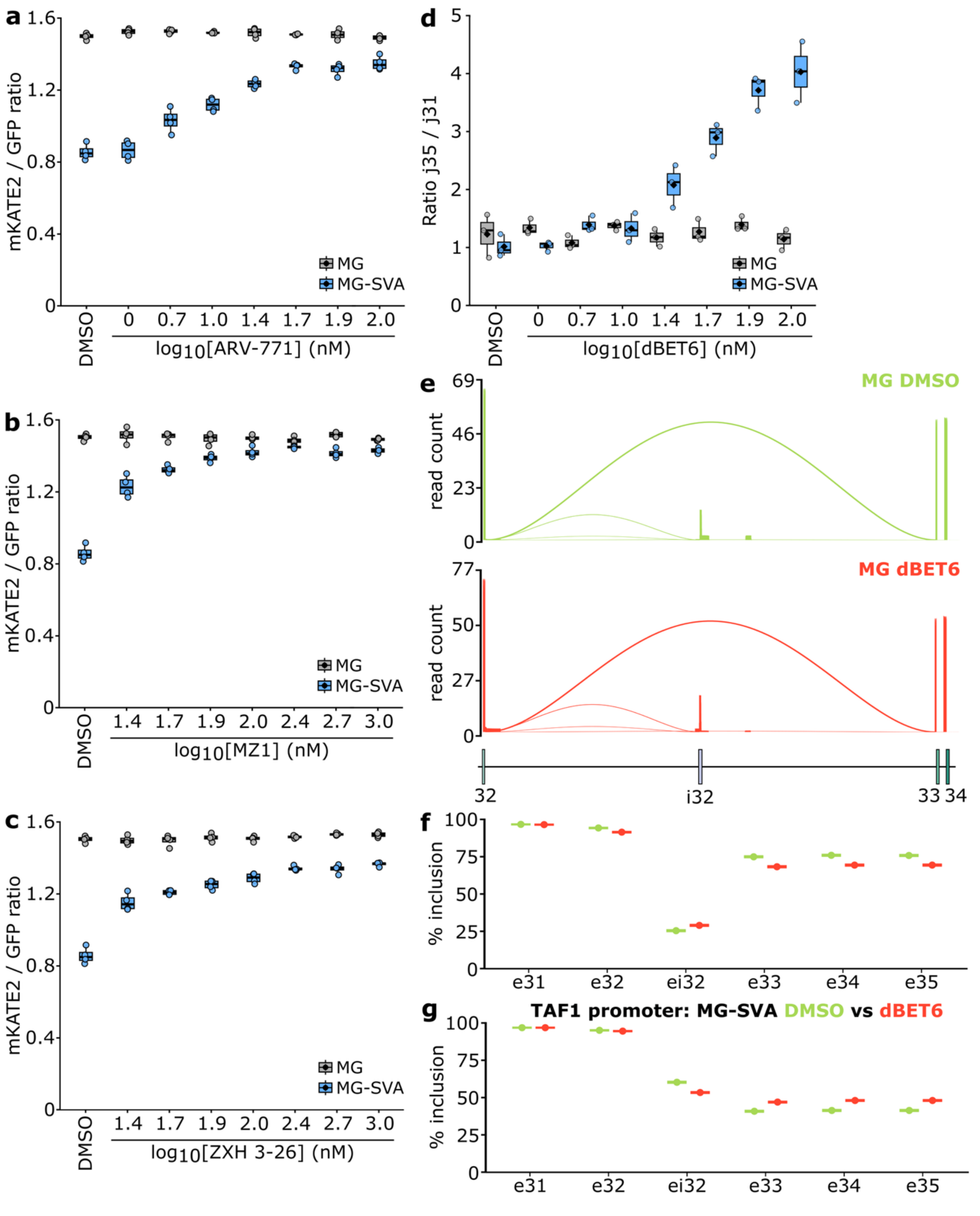

Supplementary Fig. 3: Depletion of BET proteins consistently rescues the XDP-SVA-induced misprocessing of the *TAF1* minigene reporter.

(a-c) Dose-dependent effect of ARV-771 (a), MZ1 (b) and ZXH-3-26 (c) on mKATE2/GFP ratio of fluorescence signals. Box plots generated with n=4 technical replicates. (d) Ratio of exon 35-mKATE2 to linker-exon 31 RT-qPCR products upon DMSO or dBET6 treatment. Box plots generated with n=3 biological replicates. (e) Sashimi plots depicting the usage of i32 or exon 33 acceptor sites upon DMSO or dBET6 treatment in the wild-type MG. Read count is depicted

as read counts/1000. (f-g) Dot plot (n=1) indicating the percentage of incorporation of the different minigene exons for the identified transcript isoforms upon dBET6 treatment in the wild-type MG (f) or the MG-SVA under the regulation of the TAF1 promoter (g). In f and g, error bars indicate Wilson 95% confidence intervals.

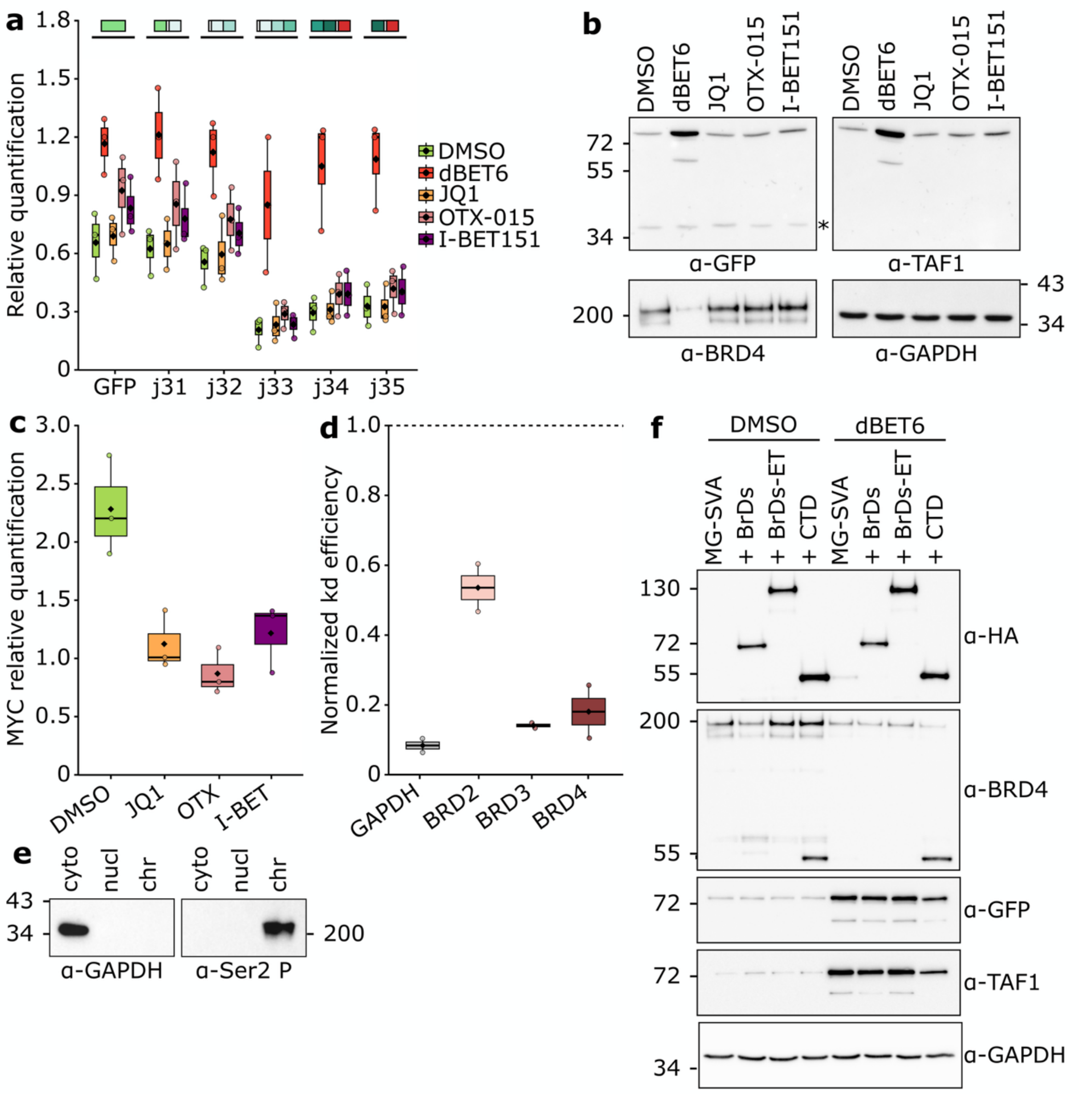

Supplementary Fig. 4: BRD4 bromodomains are dispensable for the rescue of the XDP molecular signature.

(a, b) Expansion of Fig. 3b-c including the BRD inhibitors OTX-015 and I-BET151. Box plots represent n=3 biological replicates. In b, *TAF1* minigene-derived truncated products are indicated with an asterisk (*). GAPDH is used as a dBET6-insensitive loading control. Molecular weight is expressed in kDa. (c) *MYC* relative quantification. Box plots indicate n=3 biological replicates. (d) siRNA-mediated knockdown (kd) efficiency of BRD2, BRD3 and BRD4 (corresponding to Fig. 3d) normalized against a non-targeting siRNA control. Box plots represent n=2 biological replicates. (e) Validation of chromatin fractionation purity supporting the nascent RNA analysis shown in Fig. 3e. GAPDH serves as a cytoplasmic marker, while Ser2-phosphorylated RNA pol II (Ser2 P) marks the chromatin fraction. Molecular weight is expressed in kDa. (f) Validation of BRD4 truncation constructs used in Fig. 3g. Molecular weight is expressed in kDa.

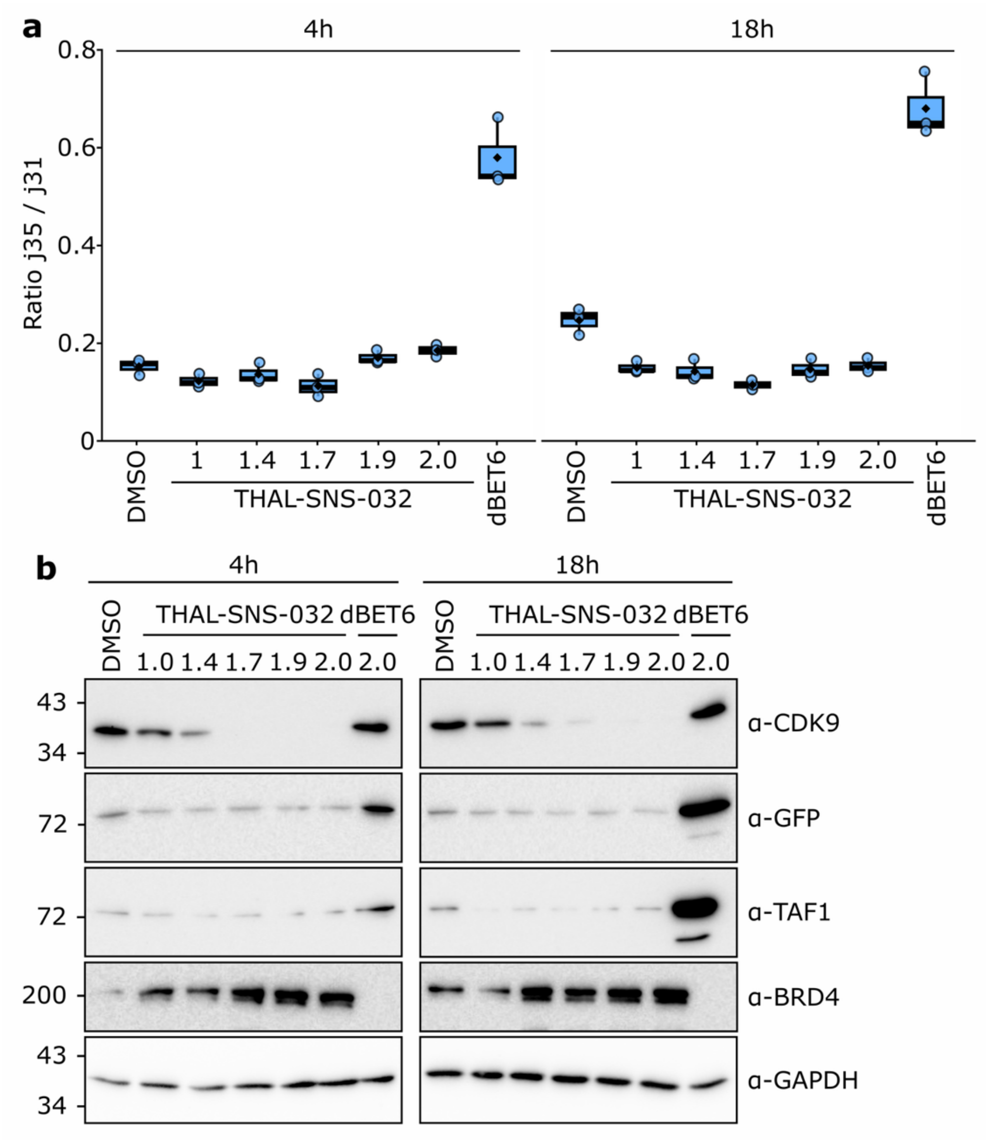

Supplementary Fig. 5: CDK9 depletion does not rescue the XDP molecular signature. (a) Quantification of *TAF1* minigene j35/j31 upon CDK9 depletion using THAL-SNS-032. Concentration of THAL-SNS-032 is expressed as log_{10}(nM). Box plots indicate n=3 technical replicates. (b) Immunoblot analysis mirroring the RT-qPCR results obtained in panel a. The compound concentrations are expressed as log_{10}(nM). Molecular weights are indicated in kDa.

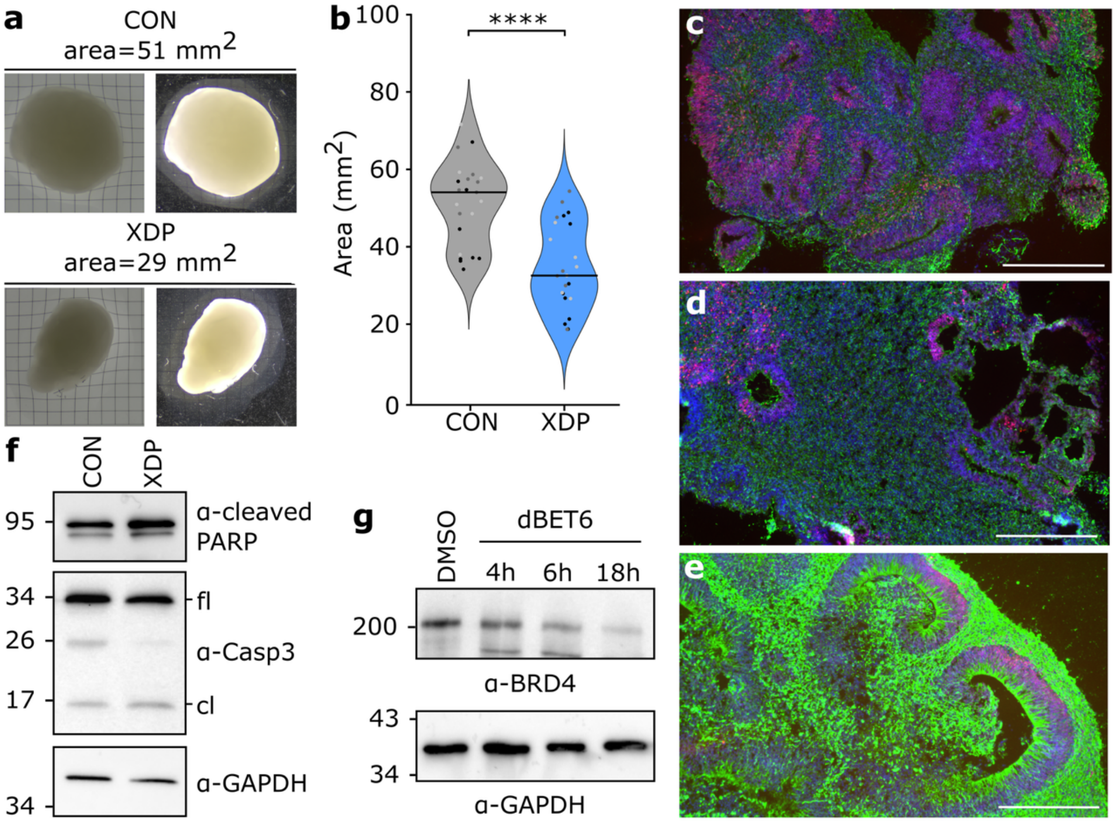

Supplementary Fig. 6: Reduced size and aberrant progenitor morphology in XDP cerebral organoids.

(a) Representative bright-field images of control and XDP-derived cerebral organoids (COs) at day 40 of differentiation. (b) Quantification of organoid cross-sectional area based on manual segmentation of organoid outlines from stereomicroscope images. Violin plots represent pooled measurements from three biological replicates (indicated in black, dark grey and light grey) using independent iPSC lines for both genotypes; p-value=7.7e-06. (c to e) Examples of rosettes morphologies observed across three biological replicates using independent XDP iPSC. Pan-neuronal marker TUJ1 in shown in green, progenitor marker PAX6 in red and nuclei are stained with DAPI. Scale bar: 300 µm. (f) Immunoblot analysis of whole-organoid lysates shows increased apoptosis in XDP organoids, as indicated by elevated cleaved PARP, mildly elevated levels of cleaved caspase-3 (cl) and reduced levels of full-length caspase-3 (fl). (g) BRD4 depletion control in XDP COs treated with 100 nM dBET6 for different time points (4 h, 6 h, and 18 h). The control relates to Fig. 5.

Supplementary Table 1: Details on the composition of the FREpi library, integration of Supplementary Figure 2a.

Target Class	Targets	Molecule counts	%
Histone Deacetylases (HDACs)	Class I/II, class III, SIRT1,2,3	142	25.7%
Other kinases	RTK, PI3K/AKT/mTOR, PKA, MAPK, BTK, JAK/STAT	54	9.8%
Histone Methyltransferases (KMTs)	EZH1/2, DOT1L, SETD2/8, MLL	52	9.4%
CDK and cell cycle kinases	CDK1/5/7/12/13, Aurora, PLK	45	8.1%
Bromodomains (BRDs)	BRD2/3/4/T, TAF1, PCAF/GCN5	44	8.0%
Protein Arginine Methyltransferases (PRMTs)	PRMT1/5/6	37	6.7%
Histone Demethylases (KDMs)	KDM4/5, LSD1	31	5.6%
DNA methyltransferases (DNMTs)	DNMT1/3	31	5.6%
Histone Acetyltransferases (HATs)	CBP/P300	30	5.4%
Chromatin remodelers	SMARCA/RUVBL1/2, EED, BCL6	24	4.3%
DNA damage response kinases (DDR)	ATM, ATR, CHK	22	4.0%
Minor, non-epigenetics	Receptors, transcription factors, agonists	18	3.3%
Protein Arginine Deaminases (PADs)	/	14	2.5%
Deubiquitinases (DUBs)	USP, OTU	10	1.8%
RNA pol II inhibitors	pol II elongation factors, transcription regulators	10	1.8%
Proteasome inhibitors	20S/26S	8	1.4%
Inactive controls	/	8	1.4%
Metabolism	Dehydrogenases, PPAR, GPCR	7	1.3%

Supplementary Table 2: Primers used in this study

Primer ID	Sequence (5'-3')	Application
GFP fw-1	AGGACGACGGCAACTACAAG	RT-qPCR minigene exons and minigene 3' RACE - PCR1
GFP rv	AAGTCGATGCCCTTCAGCTC	RT-qPCR minigene exons
GFP fw-2	GGATCACTCTCGGCATGGAC	RT-qPCR minigene exons
j31 rv	TGGGTGATGAAATGGGAAGCC	RT-qPCR minigene exons
Linker fw	GGCTTCCCATTTCATCACCC	RT-qPCR minigene exons
j32 rv	TGTGCTTGGAGATGTTCTTACG	RT-qPCR minigene exons
j33 rv	AGTATACTGACTCTCAGGTCC	RT-qPCR minigene exons
TAF1 ex 34 fw	CCTCAGCCTCCTGATTTGTA	RT-qPCR minigene exons
TAF1 ex 35 fw	TGATACCAACACATCCCTCAGT	RT-qPCR minigene exons
mKATE2 linker rv	CTGGATCGAACCACTTTGTAC	RT-qPCR minigene exons - for dual color
TAF1 ex 35 rv	TTACTGTGTTACCTGCTTTTCTG	RT-qPCR minigene exons for single color
i32 fw	TCGGGAGAGCTTTCTGGATG	RT-qPCR intronic exon i32
i32 rv	TGAAGGAAGCTGAGTGAGAAG	RT-qPCR intronic exon i32
GAPDH fw	TGCACCACCAACTGCTTAGC	RT-qPCR reference genes
GAPDH rv	GGCATGGACTGTGGTCATGAG	RT-qPCR reference genes
ACTN fw	CACCATTGGCAATGAGCGGTTC	RT-qPCR reference genes
ACTN rv	AGGTCTTTGCGGATGTCCACGT	RT-qPCR reference genes
3' RACE MG fw 2	GGCTTCCCATTTCATCACCC	Minigene 3' RACE - PCR2
BRD2 fw	GAGGTGTCCAATCCCAAAAAGC	RT-qPCR
BRD2 rv	ATGCGAACTGATGTTTCCACA	RT-qPCR
BRD3 fw	TCAAATTGAACCTGCCGGATT	RT-qPCR
BRD3 rv	TGCATACATTCGCTTGCACTC	RT-qPCR
BRD4 fw	TCCTGACCATGAGGTGGTG	RT-qPCR
BRD4 rv	CAAAGCGCATTTCGAACAC	RT-qPCR
5'-scar fw	AAATGCAGCTTGGAACCAGG	RT-qPCR on minigene nascent RNA
5'-scar rv	AATATGGGAGACAGGAGTGGG	RT-qPCR on minigene nascent RNA
3'-scar fw	TAGCACGTCCACCATCTCAA	RT-qPCR on minigene nascent RNA
3'-scar rv	TCCTGGCCTTAAGTGATCCG	RT-qPCR on minigene nascent RNA
GAPDH fw	AATCCCATCACCATCTTCCAG	RT-qPCR on reference gene nascent RNA
GAPDH rv	GAGCCACACCATCCTAGTTG	RT-qPCR on reference gene nascent RNA
MYC fw	GGCTCCTGGCAAAAGGTCA	RT-qPCR
MYC rv	CTGCGTAGTTGTGCTGATGT	RT-qPCR
3' RACE XDP fw 1	AGCTATCAACCCCTTGCTGG	3' RACE on PFCs and COs
3' RACE XDP fw 2	GGCGTTTTCTTTCATTCTGG	3' RACE on PFCs and COs
