# Supplementary material for: Correction of the molecular phenotype of X-linked Dystonia-Parkinsonism reveals a non-canonical function of BRD4
Source: Nat Commun. 2026 May 5;17:4062. doi: 10.1038/s41467-026-72319-6 (PMC13144358; doi:10.1038/s41467-026-72319-6)
Supplement: Supplementary file 2 — Reporting Summary [file 41467_2026_72319_MOESM2_ESM.pdf]

nature portfolio

Corresponding author(s): Prof. Dr. Marc Timmers

Last updated by author(s): Mar 10, 2026

Reporting Summary

Nature Portfolio wishes to improve the reproducibility of the work that we publish. This form provides structure for consistency and transparency in reporting. For further information on Nature Portfolio policies, see our Editorial Policies and the Editorial Policy Checklist.

Statistics

For all statistical analyses, confirm that the following items are present in the figure legend, table legend, main text, or Methods section.

n/a	Confirmed	
☐	☒	The exact sample size (n) for each experimental group/condition, given as a discrete number and unit of measurement
☐	☒	A statement on whether measurements were taken from distinct samples or whether the same sample was measured repeatedly
☐	☒	The statistical test(s) used AND whether they are one- or two-sided<br>*Only common tests should be described solely by name; describe more complex techniques in the Methods section.*
☒	☐	A description of all covariates tested
☐	☒	A description of any assumptions or corrections, such as tests of normality and adjustment for multiple comparisons
☐	☒	A full description of the statistical parameters including central tendency (e.g. means) or other basic estimates (e.g. regression coefficient) AND variation (e.g. standard deviation) or associated estimates of uncertainty (e.g. confidence intervals)
☐	☒	For null hypothesis testing, the test statistic (e.g. F, t, r) with confidence intervals, effect sizes, degrees of freedom and P value noted<br>*Give P values as exact values whenever suitable.*
☒	☐	For Bayesian analysis, information on the choice of priors and Markov chain Monte Carlo settings
☒	☐	For hierarchical and complex designs, identification of the appropriate level for tests and full reporting of outcomes
☒	☐	Estimates of effect sizes (e.g. Cohen's d, Pearson's r), indicating how they were calculated

Our web collection on statistics for biologists contains articles on many of the points above.

Software and code

Policy information about availability of computer code

Data collection	Long-read sequencing data has been collected using MinKnow (v. 24.11.10)
Data analysis	RT-qPCR analysis and screening data have been analysed using custom code in RStudio (v. 2022.12.0+353). Long-read sequencing data analysis has been performed using Dorado (v. 0.7.1) for basecalling, Guppy for demultiplexing and minimap2 (v. 2.28-r1209) for alignment. Subsequent analysis has been carried out using custom R and Python (v. 3.11.8) scripts as defined in the Methods section.

For manuscripts utilizing custom algorithms or software that are central to the research but not yet described in published literature, software must be made available to editors and reviewers. We strongly encourage code deposition in a community repository (e.g. GitHub). See the Nature Portfolio guidelines for submitting code & software for further information.

Data

Policy information about availability of data

All manuscripts must include a data availability statement. This statement should provide the following information, where applicable:

- Accession codes, unique identifiers, or web links for publicly available datasets
- A description of any restrictions on data availability
- For clinical datasets or third party data, please ensure that the statement adheres to our policy

Long-read sequencing data has been deposited at the Sequence Read Archive (SRA) under the ID: PRJNA1306395.

Research involving human participants, their data, or biological material

Policy information about studies with human participants or human data. See also policy information about sex, gender (identity/presentation), and sexual orientation and race, ethnicity and racism.

Reporting on sex and gender	Human data presented in the manuscript refers to the generation and characterization of cerebral organoids derived from human induced-pluripotent stem cells. Here, we have used male cell lines, as indicated in the Methods section. The term "male" is related to the biological sex of the donors, as the disease is X-linked.
Reporting on race, ethnicity, or other socially relevant groupings	All XDP and control donors were of Filipino ancestry, consistent with the known population distribution of the disease.
Population characteristics	The study used hiPSC lines derived from XDP patients and unaffected control donors and post-mortem specimens from XDP patinets
Recruitment	Patient samples were obtained from previously consented donors through the Collaborative Center for X-linked Dystonia Parkinsonism (CC-XDP) at Massachusetts General Hospital in Boston (MA, USA).
Ethics oversight	Research involving human material complied with relevant ethical regulations at our institutions and was specifically evaluated and approved by the review board of Massachusetts General Hospital (Boston, MA, USA) for generation and distribution. Written informed consent for the use of donated material for research purposes was obtained from all donors.

Note that full information on the approval of the study protocol must also be provided in the manuscript.

Field-specific reporting

Please select the one below that is the best fit for your research. If you are not sure, read the appropriate sections before making your selection.

[x] Life sciences [ ] Behavioural & social sciences [ ] Ecological, evolutionary & environmental sciences

For a reference copy of the document with all sections, see nature.com/documents/nr-reporting-summary-flat.pdf

Life sciences study design

All studies must disclose on these points even when the disclosure is negative.

Sample size	No statistical power calculation was performed. For cerebral organoid experiments, we used n = 3 independent organoids per genotype and per condition, generated from three independent differentiations of distinct iPSC lines. This sample size is consistent with common practice in the field and was chosen to capture the physiological variability intrinsic to organoid systems while enabling reproducible detection of robust phenotypes. For cell culture–based assays, replicate numbers (typically n = 3 biological replicates) were similarly chosen based on established practice in the field.
Data exclusions	No data were excluded from the analyses.
Replication	All key experiments were independently repeated two or three times with consistent results. For organoid experiments, findings were reproduced across independent differentiations and distinct iPSC lines. For cell-based reporter assays, results were replicated across different cell lines (HeLa, 293T, U-2 OS) and under independent experimental conditions. For RT-qPCR experiments, we use n = 3 technical replicates and for the compound screening we used n= 4 technical replicates.
Randomization	Randomization was not applicable for cell culture and organoid experiments, as group allocation was based on genotype (control vs. XDP) and experimental condition (e.g., compound treatment vs. DMSO). Within each experiment, all conditions were processed in parallel using identical protocols to minimize technical bias.
Blinding	Investigators were not blinded during sample collection because genotypes and treatments were evident during handling. However, data acquisition and analysis were performed using automated pipelines (e.g., qPCR quantification, imaging-based readouts, sequencing analyses) to minimize operator bias.

Reporting for specific materials, systems and methods

We require information from authors about some types of materials, experimental systems and methods used in many studies. Here, indicate whether each material, system or method listed is relevant to your study. If you are not sure if a list item applies to your research, read the appropriate section before selecting a response.

Materials & experimental systems

n/a	Involved in the study
☐	☒ Antibodies
☐	☒ Eukaryotic cell lines
☒	☐ Palaeontology and archaeology
☒	☐ Animals and other organisms
☒	☐ Clinical data
☒	☐ Dual use research of concern
☒	☐ Plants

Methods

n/a	Involved in the study
☒	☐ ChIP-seq
☒	☐ Flow cytometry
☒	☐ MRI-based neuroimaging

Antibodies

Antibodies used	The antibody used included: GFP (Takara, cat numb 632381, clone ID JL8, lot numb A8034133, 1:2000), TAF1 (from Capponi et al., RNA Biol, 2020, 1:1000), GAPDH (Millipore, cat numb MAB374 , clone ID 6C5, lot numb 4047566, 1:1500), BRD4 (Cell Signaling, cat numb 13440, clone ID E2A7X, lot numb 10, 1:1000), Vinculin (Santa Cruz, cat numb sc-73614, clone ID 7F9, lot numb H1721, 1:1000), CDK9 (Cell Signaling, cat numb 2316, clone ID C12F7, lot numb 10, 1:1000), pol II Ser2-P (Cell Signaling, cat numb 31262, clone ID E1Z3G, lot numb 1, 1:1000), cleaved PARP (Cell Signaling, cat numb 9541, clone ID N/A, lot numb 22, 1:1000), Caspase 3 (Cell Signaling, cat numb 9662, clone ID N/A, lot numb 19, 1:500), PAX6 (Invitrogen, cat. no. 42-6600, lot YL388278, 1:100), TUJ1 (R&D Systems, cat. no. MAB 1195, lot HGQ0421032, 1:500) and TBR2 (Abcam, cat. no. ab23345, clone ID EPR19012, 1:500).
Validation	GFP antibody: specificity for GFP-tagged proteins confirmed by manufacturer; BRD4 antibody: specificity confirmed by BRD4 protac (Figure 3c); CDK9 antibody: specificity confirmed by CDK9 protac (Supplementary Figure 2); cleaved PARP antibody: specificity confirmed by manufacturer upon staurosporine treatment; caspase 3 antibody: specificity confirmed by manufacturer upon caspase 3 knock-out.

Eukaryotic cell lines

Policy information about cell lines and Sex and Gender in Research

Cell line source(s)	Flp-In T-REx HeLa cells were a kind gift from Dr. G. Kops, Hubrecht Institute, NL, Flp-In T-REx 293 cells were obtained from Thermo Fisher Scientific (cat. no. R78007) and Flp-In T-REx U-2 OS were a kind gift from Dr. K. Haynes, Arizona State University, USA. hiPSCs derived from three XDP male patients (32517, 35833 and 34363) and three male controls (33113, 33114 and 33362) were obtained via the Collaborative Center for X-linked Dystonia Parkinsonism at Massachusetts General Hospital in Boston (MA, USA).
Authentication	None of the cell line used were authenticated.
Mycoplasma contamination	Flp-In T-REx HeLa, 293T and U-2 OS cell lines were tested for mycoplasma contamination and resulted negative. hiPSCs were not tested for mycoplasma contamination by PCR methgods, however DAPI staining failed to detect cytoplasmic signal, indicative of mycoplasma contamination.
Commonly misidentified lines (See ICLAC register)	None of the cell lines used is listed in the ICLAC register (v. 13) as commonly misidentified line.

Plants

Seed stocks	*Report on the source of all seed stocks or other plant material used. If applicable, state the seed stock centre and catalogue number. If plant specimens were collected from the field, describe the collection location, date and sampling procedures.*
Novel plant genotypes	*Describe the methods by which all novel plant genotypes were produced. This includes those generated by transgenic approaches, gene editing, chemical/radiation-based mutagenesis and hybridization. For transgenic lines, describe the transformation method, the number of independent lines analyzed and the generation upon which experiments were performed. For gene-edited lines, describe the editor used, the endogenous sequence targeted for editing, the targeting guide RNA sequence (if applicable) and how the editor was applied.*
Authentication	*Describe any authentication procedures for each seed stock used or novel genotype generated. Describe any experiments used to assess the effect of a mutation and, where applicable, how potential secondary effects (e.g. second site T-DNA insertions, mosiacism, off-target gene editing) were examined.*
