# Supplementary figures and images for: Correction of the molecular phenotype of X-linked Dystonia-Parkinsonism reveals a non-canonical function of BRD4
Source: Nat Commun. 2026 May 5;17:4062. doi: 10.1038/s41467-026-72319-6 (PMC13144358; doi:10.1038/s41467-026-72319-6)

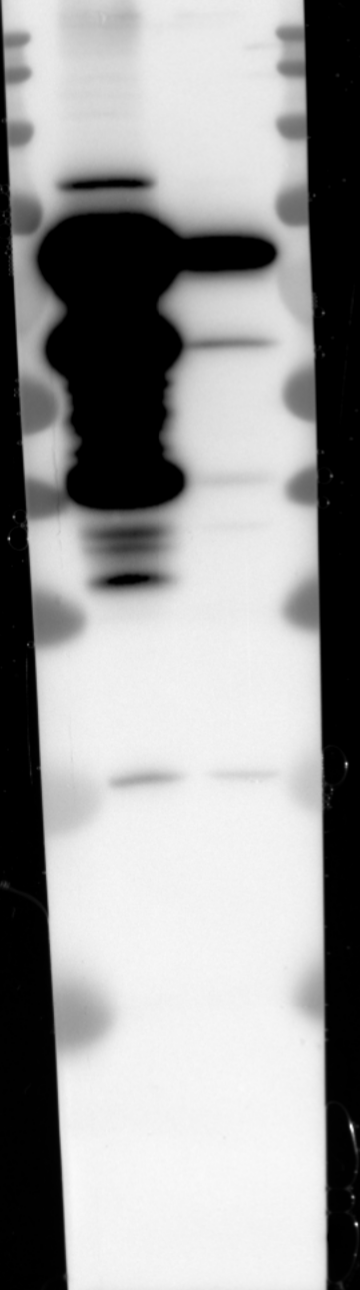

Supplement: Supplementary file 4 — Source data [file 41467_2026_72319_MOESM4_ESM.zip › Source Data/Uncropped immunoblots/Fig.1g_TAF1_long.png]

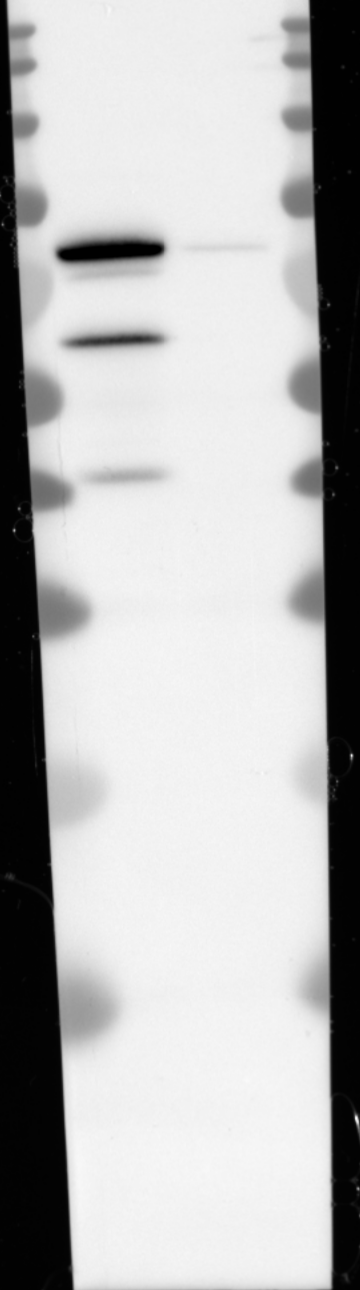

Supplement: Supplementary file 4 — Source data [file 41467_2026_72319_MOESM4_ESM.zip › Source Data/Uncropped immunoblots/Fig.1g_TAF1_short.png]

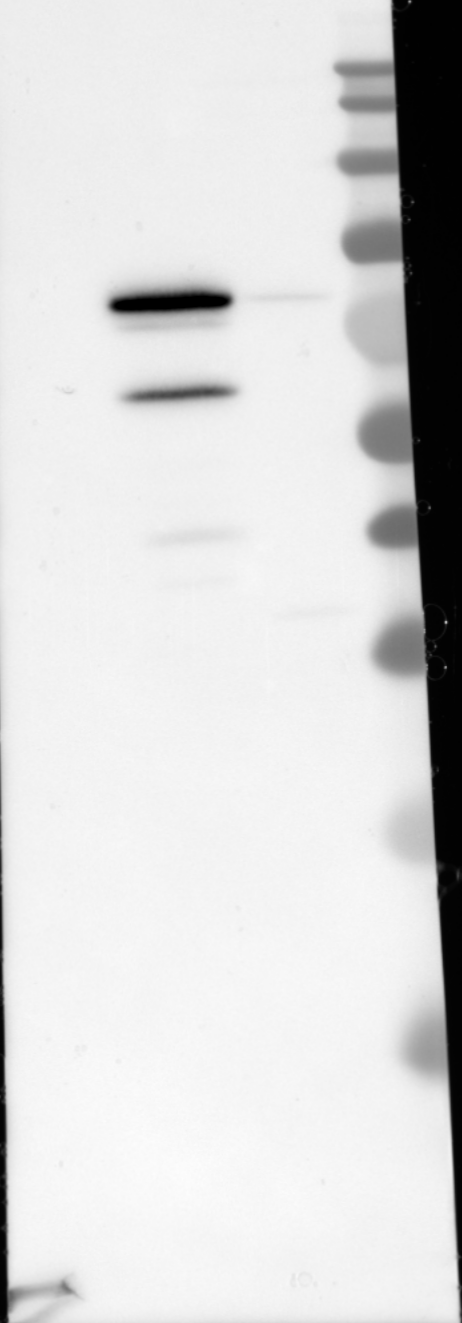

Supplement: Supplementary file 4 — Source data [file 41467_2026_72319_MOESM4_ESM.zip › Source Data/Uncropped immunoblots/Fig.1g_GFP_short.png]

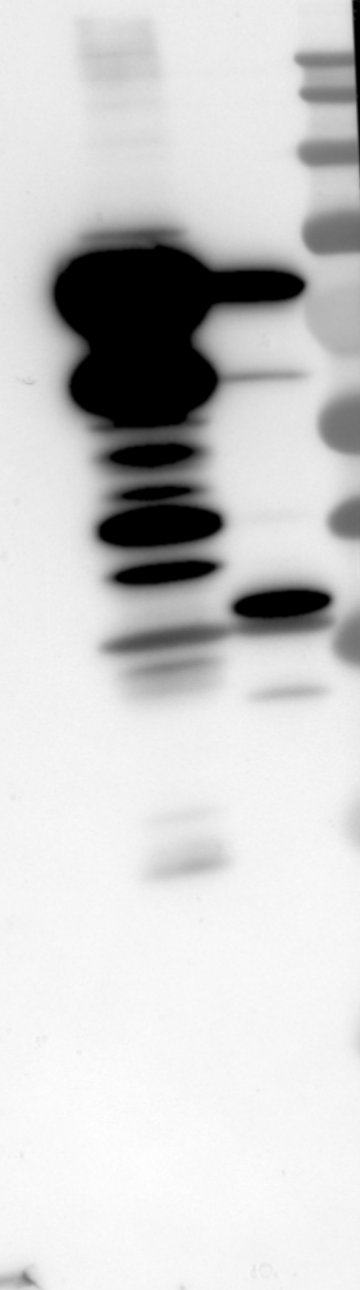

Supplement: Supplementary file 4 — Source data [file 41467_2026_72319_MOESM4_ESM.zip › Source Data/Uncropped immunoblots/Fig.1g_GFP_long.png]

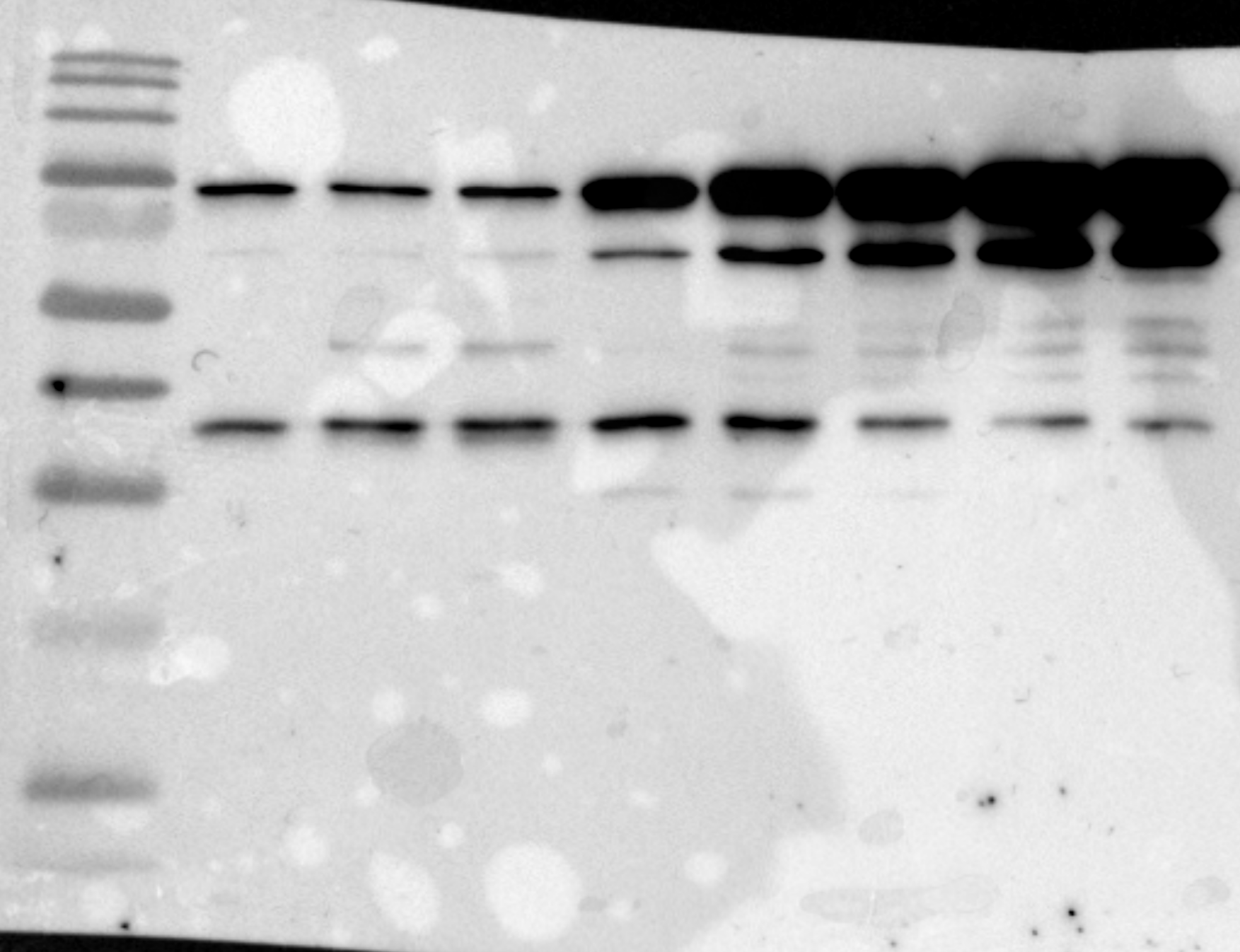

Supplement: Supplementary file 4 — Source data [file 41467_2026_72319_MOESM4_ESM.zip › Source Data/Uncropped immunoblots/Fig.2c_GFP_long.png]

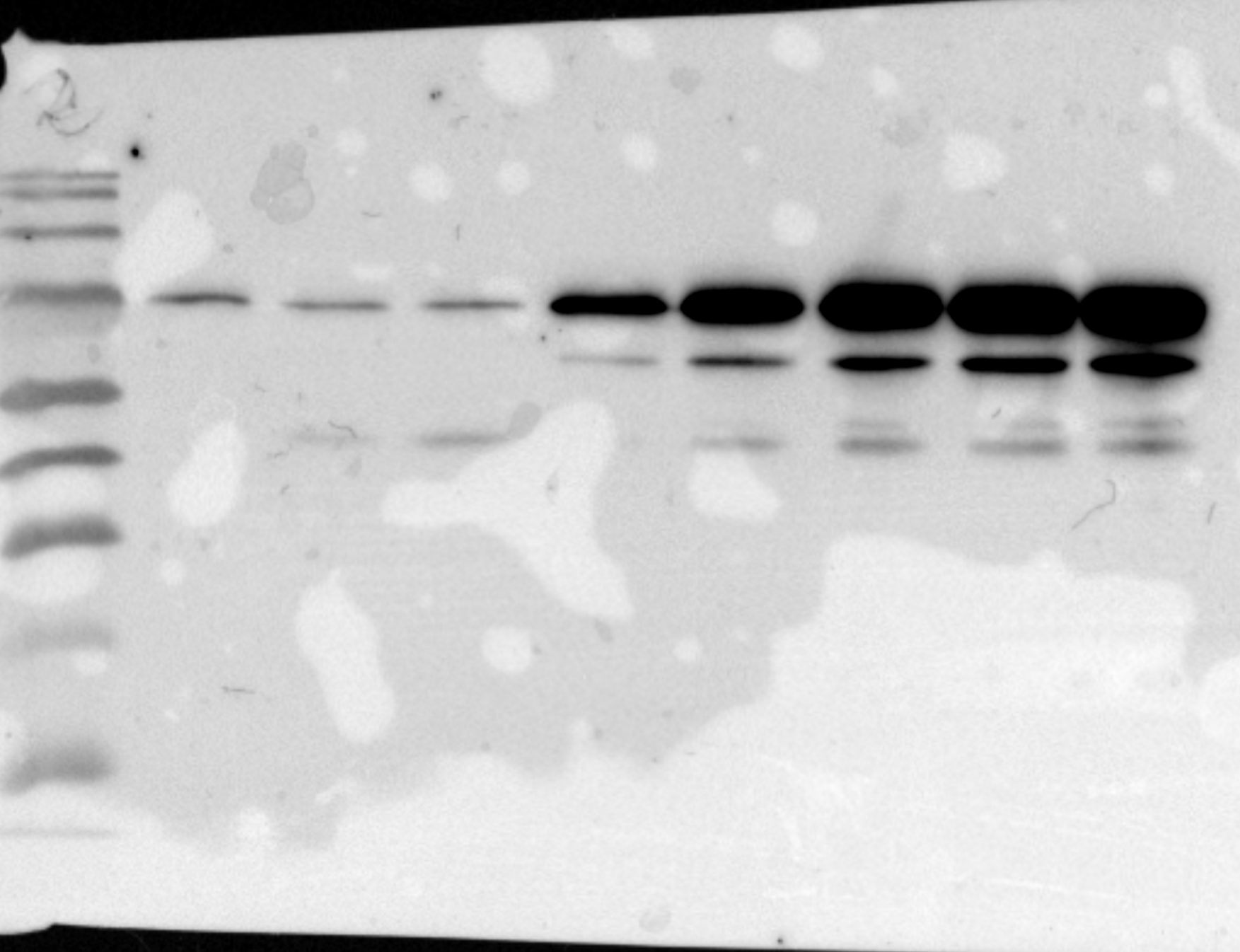

Supplement: Supplementary file 4 — Source data [file 41467_2026_72319_MOESM4_ESM.zip › Source Data/Uncropped immunoblots/Fig.2c_TAF1_long.png]

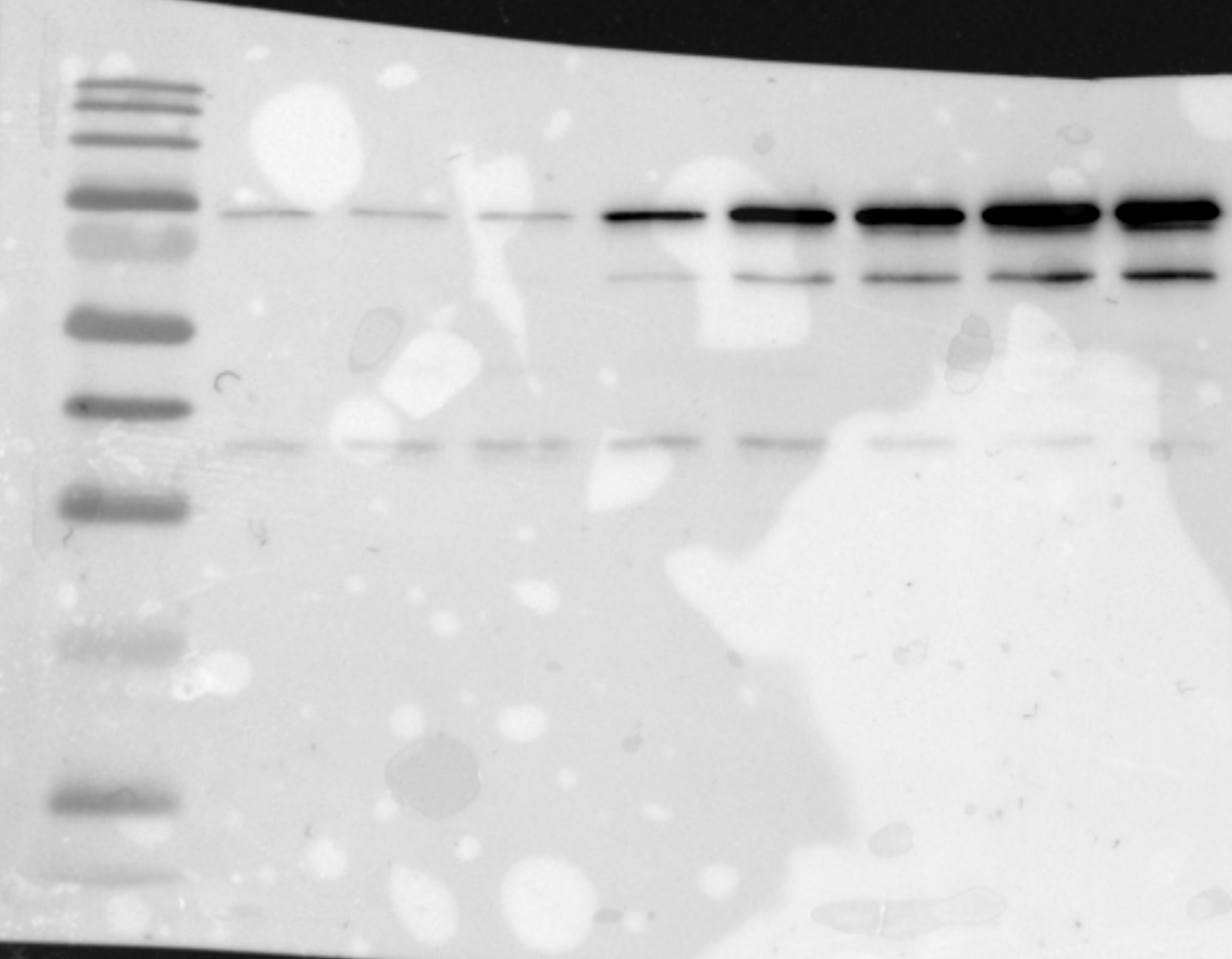

Supplement: Supplementary file 4 — Source data [file 41467_2026_72319_MOESM4_ESM.zip › Source Data/Uncropped immunoblots/Fig.2c_GFP_short.png]

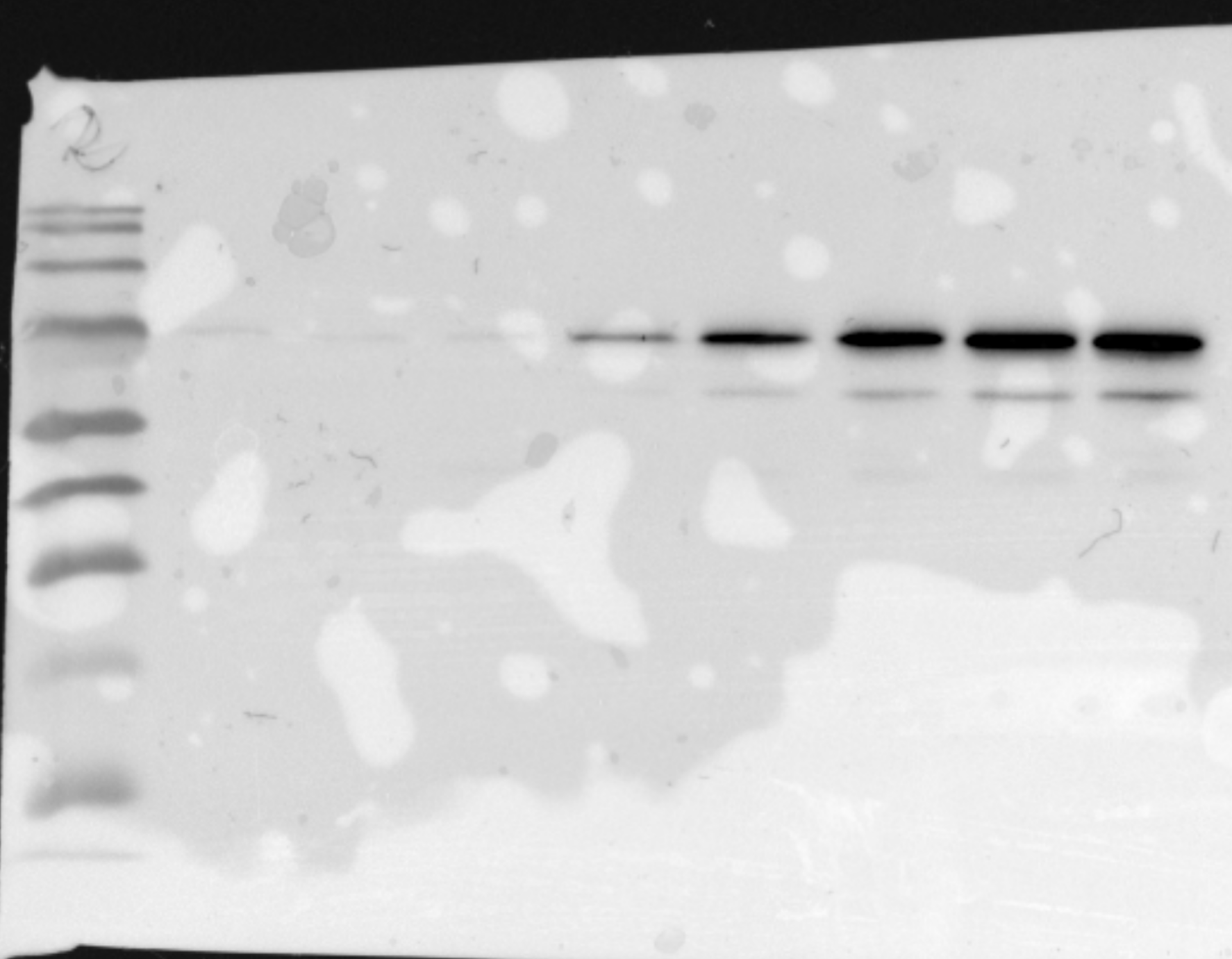

Supplement: Supplementary file 4 — Source data [file 41467_2026_72319_MOESM4_ESM.zip › Source Data/Uncropped immunoblots/Fig.2c_TAF1_short.png]

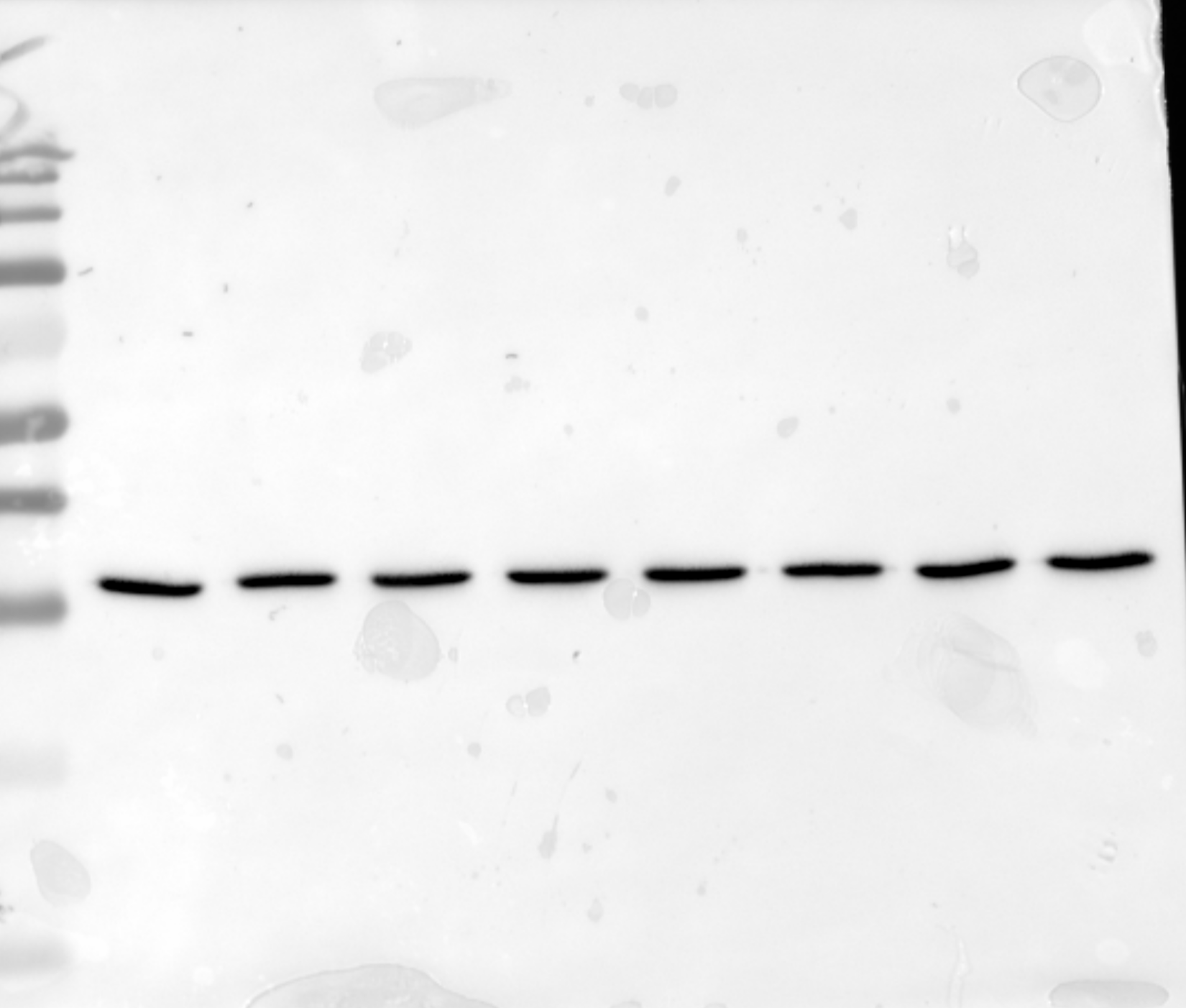

Supplement: Supplementary file 4 — Source data [file 41467_2026_72319_MOESM4_ESM.zip › Source Data/Uncropped immunoblots/Fig.2c_GAPDH.png]

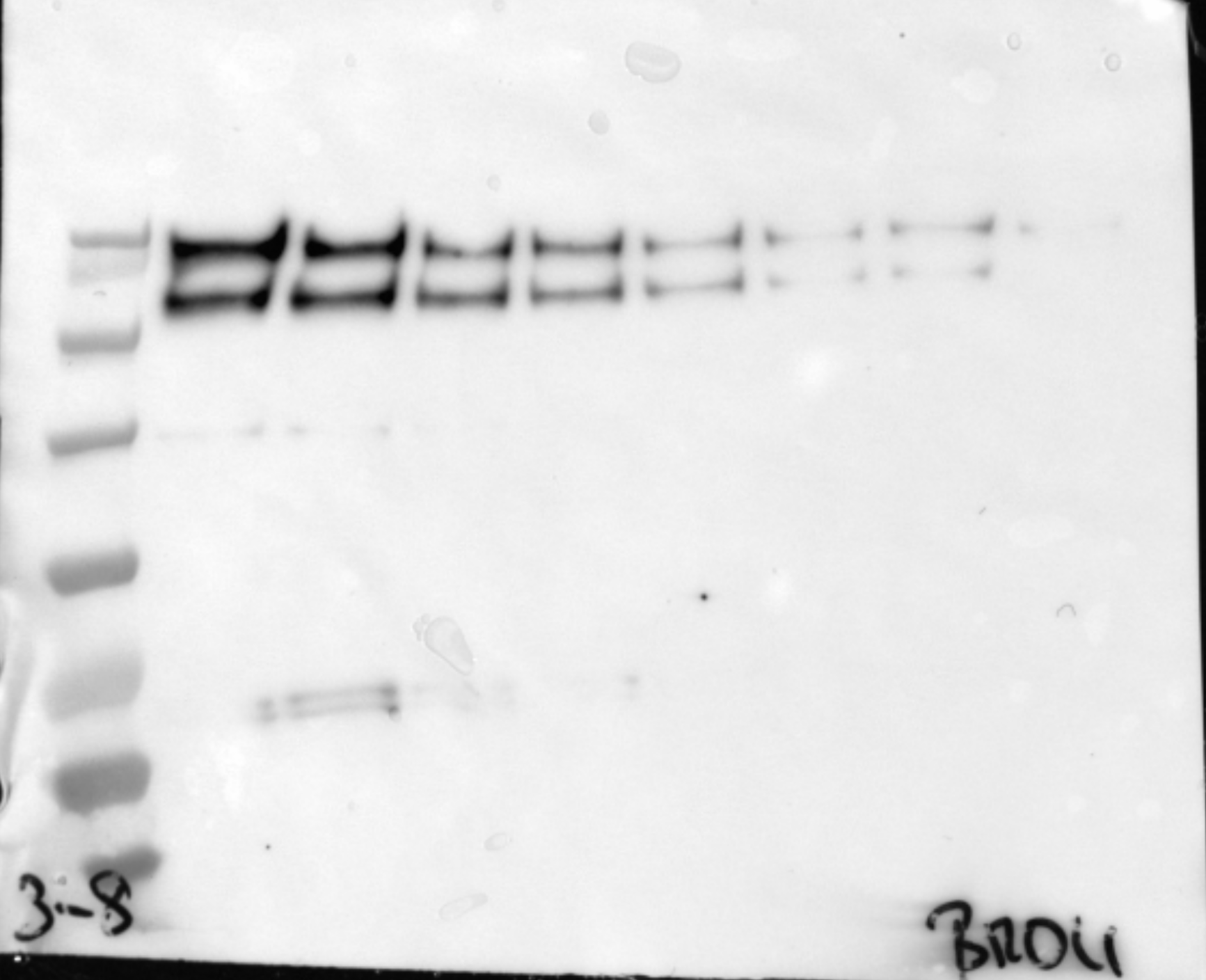

Supplement: Supplementary file 4 — Source data [file 41467_2026_72319_MOESM4_ESM.zip › Source Data/Uncropped immunoblots/Fig.2c_BRD4.png]

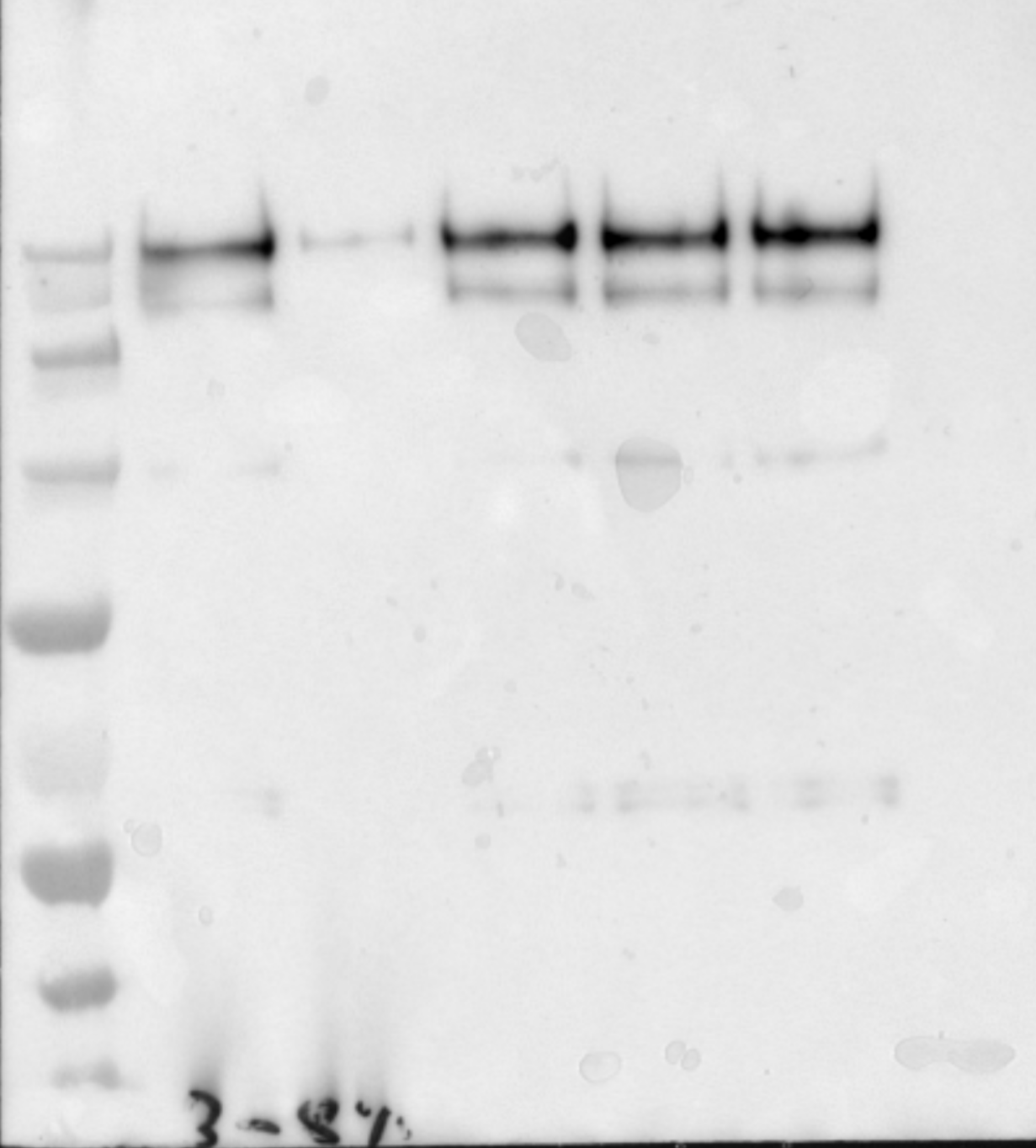

Supplement: Supplementary file 4 — Source data [file 41467_2026_72319_MOESM4_ESM.zip › Source Data/Uncropped immunoblots/Suppl. Fig.4b_BRD4.png]

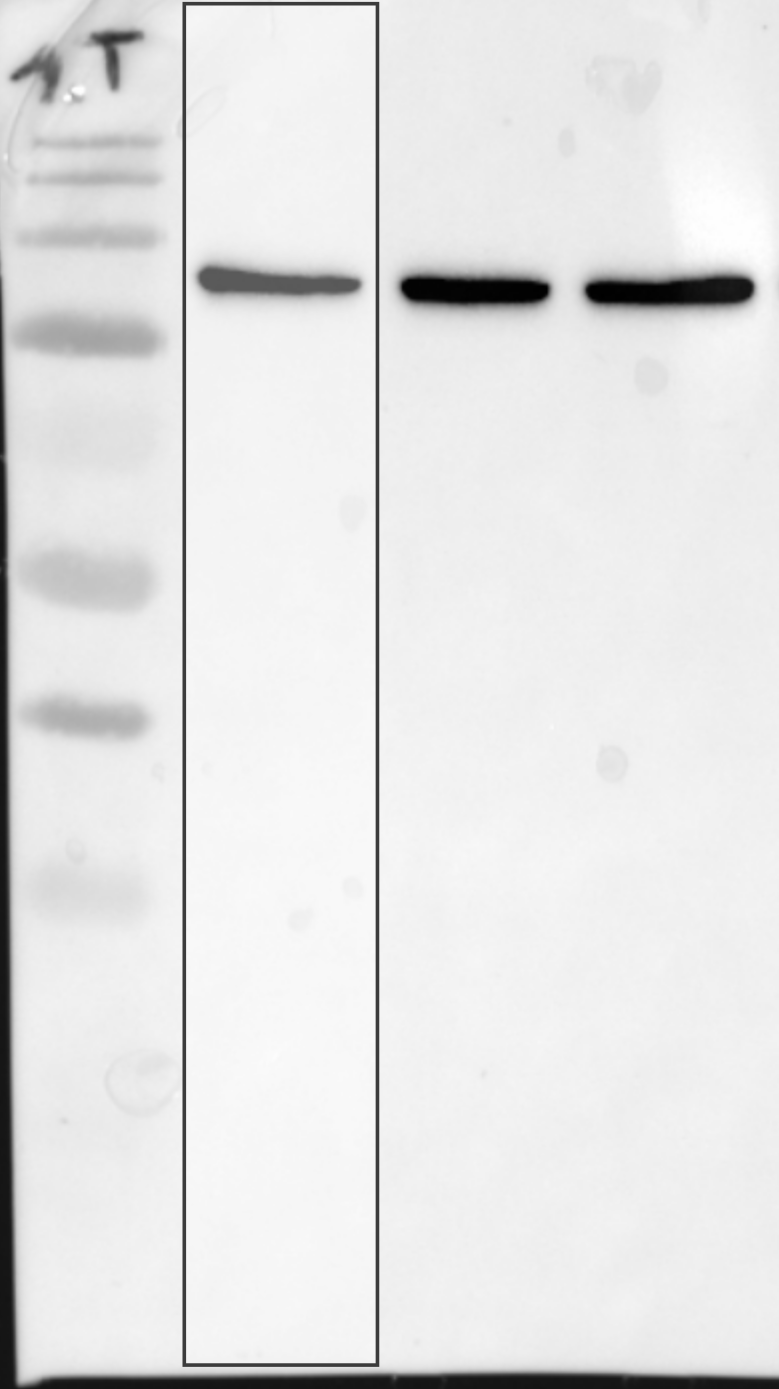

Supplement: Supplementary file 4 — Source data [file 41467_2026_72319_MOESM4_ESM.zip › Source Data/Uncropped immunoblots/Suppl. Fig.1i_VINCULIN.png]

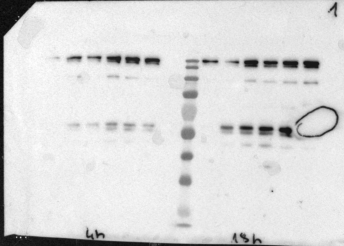

Supplement: Supplementary file 4 — Source data [file 41467_2026_72319_MOESM4_ESM.zip › Source Data/Uncropped immunoblots/Suppl. Fig.5b_BRD4.png]

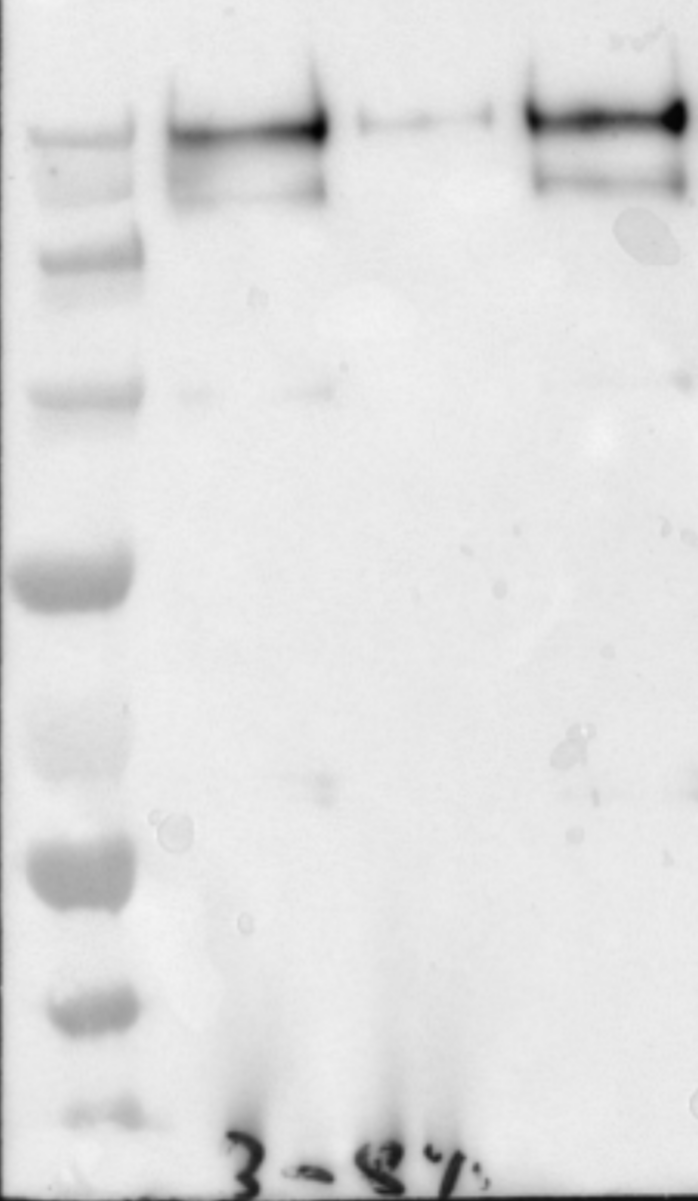

Supplement: Supplementary file 4 — Source data [file 41467_2026_72319_MOESM4_ESM.zip › Source Data/Uncropped immunoblots/Fig.3c_BRD4.png]

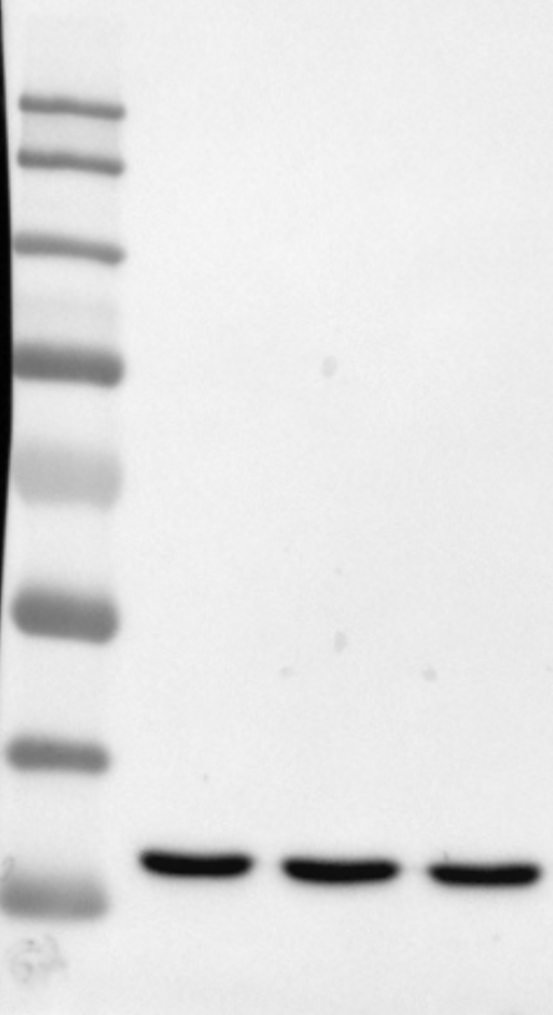

Supplement: Supplementary file 4 — Source data [file 41467_2026_72319_MOESM4_ESM.zip › Source Data/Uncropped immunoblots/Fig.3c_GAPDH.png]

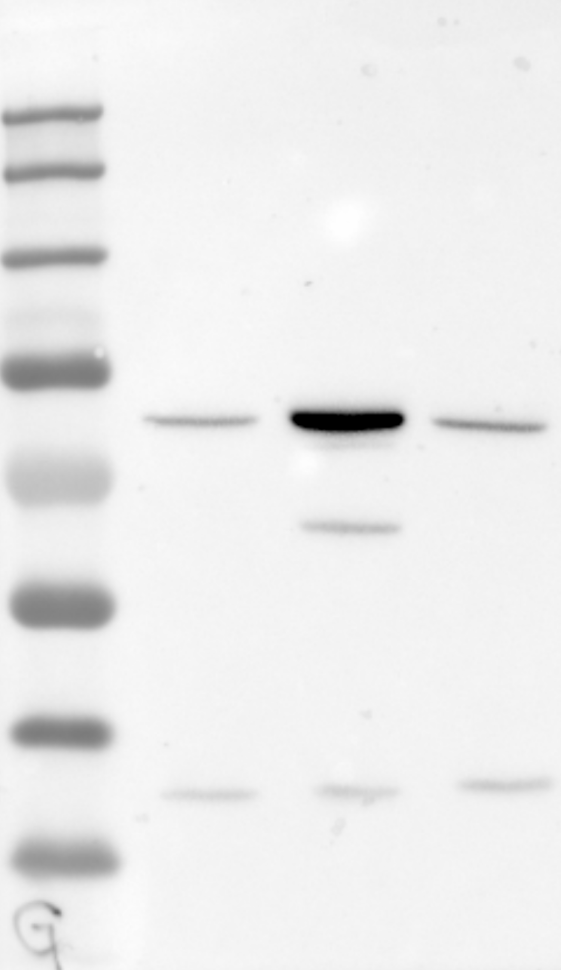

Supplement: Supplementary file 4 — Source data [file 41467_2026_72319_MOESM4_ESM.zip › Source Data/Uncropped immunoblots/Fig.3c_GFP.png]

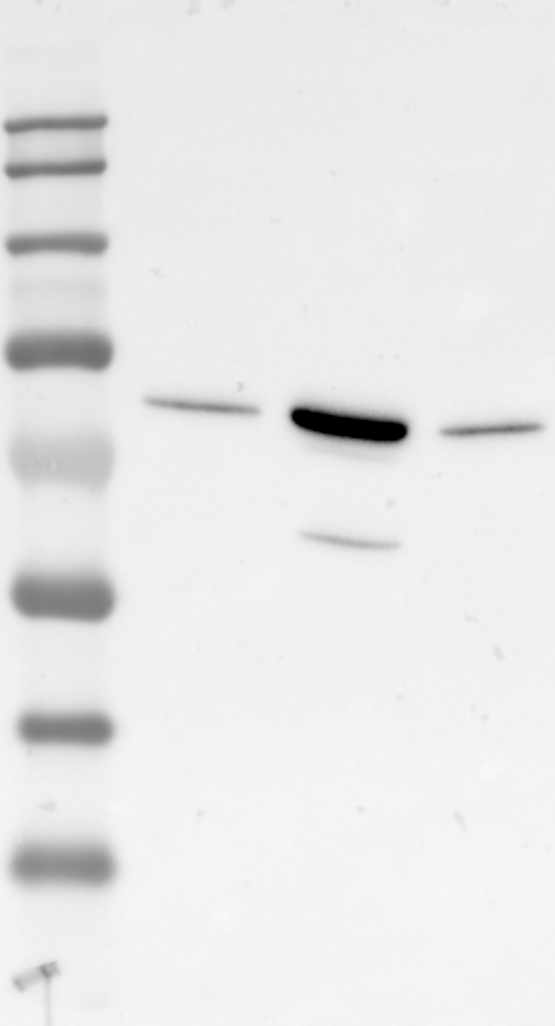

Supplement: Supplementary file 4 — Source data [file 41467_2026_72319_MOESM4_ESM.zip › Source Data/Uncropped immunoblots/Fig.3c_TAF1.png]

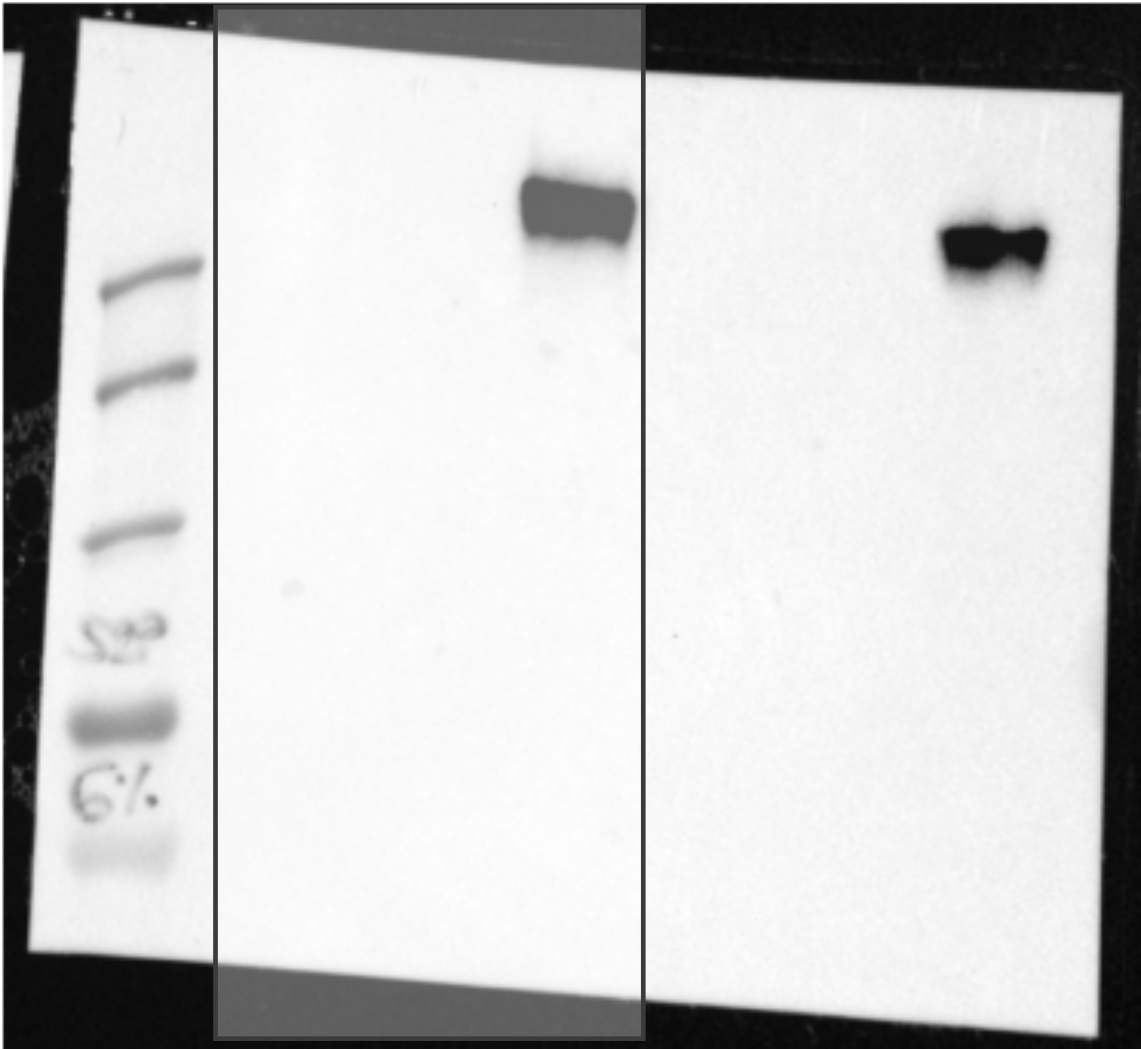

Supplement: Supplementary file 4 — Source data [file 41467_2026_72319_MOESM4_ESM.zip › Source Data/Uncropped immunoblots/Suppl. Fig.4e_Ser2P.png]

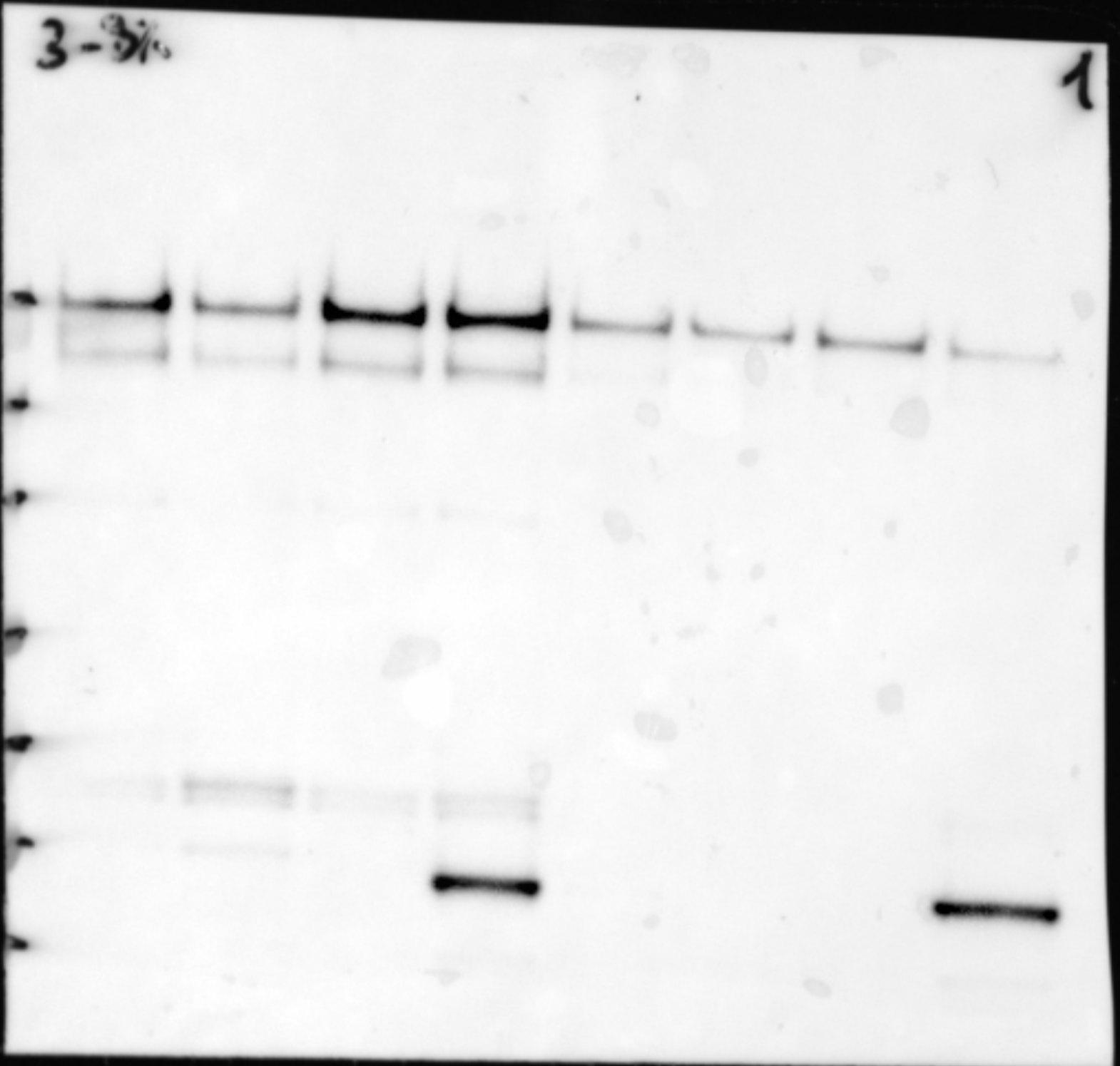

Supplement: Supplementary file 4 — Source data [file 41467_2026_72319_MOESM4_ESM.zip › Source Data/Uncropped immunoblots/Suppl. Fig.4f_BRD4.png]

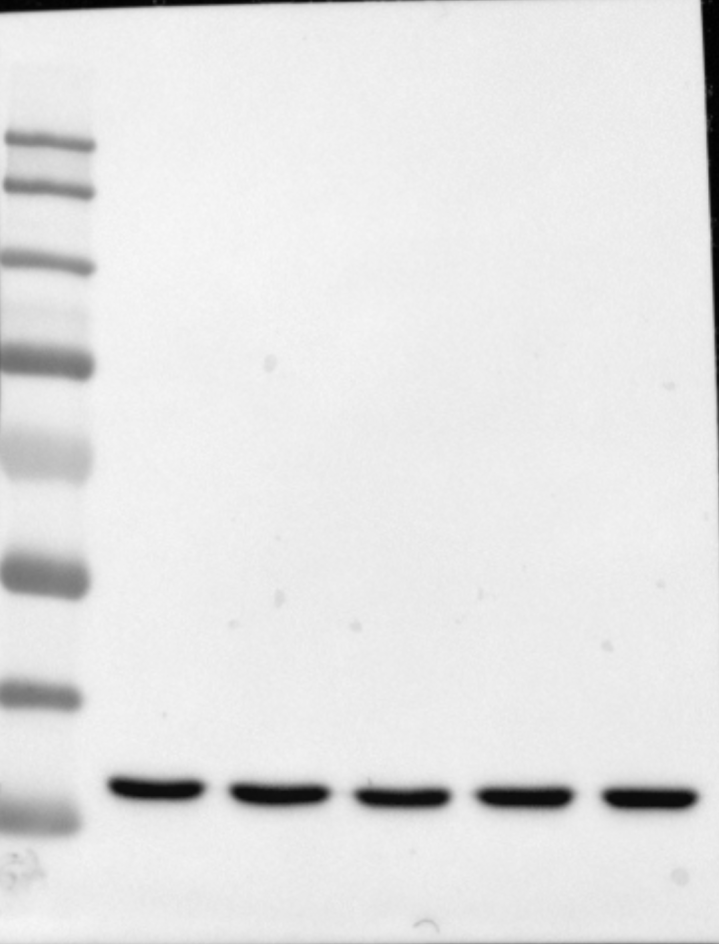

Supplement: Supplementary file 4 — Source data [file 41467_2026_72319_MOESM4_ESM.zip › Source Data/Uncropped immunoblots/Suppl. Fig.4b_GAPDH.png]

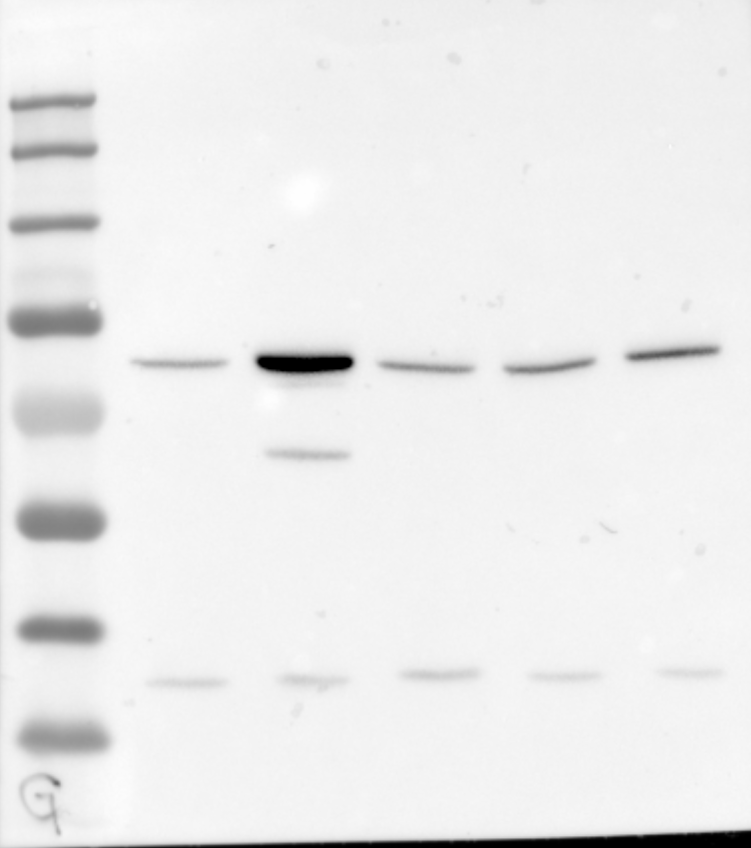

Supplement: Supplementary file 4 — Source data [file 41467_2026_72319_MOESM4_ESM.zip › Source Data/Uncropped immunoblots/Suppl. Fig.4b_GFP.png]

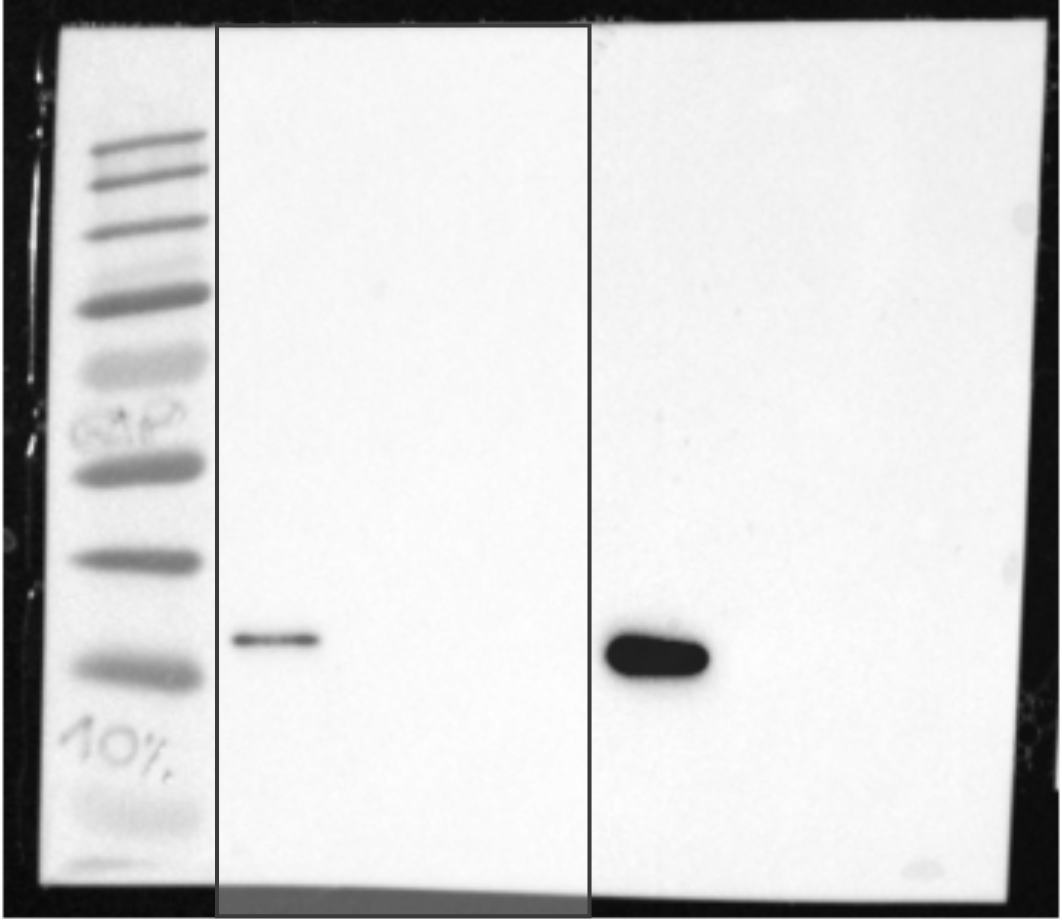

Supplement: Supplementary file 4 — Source data [file 41467_2026_72319_MOESM4_ESM.zip › Source Data/Uncropped immunoblots/Suppl. Fig.4e_GAPDH.png]

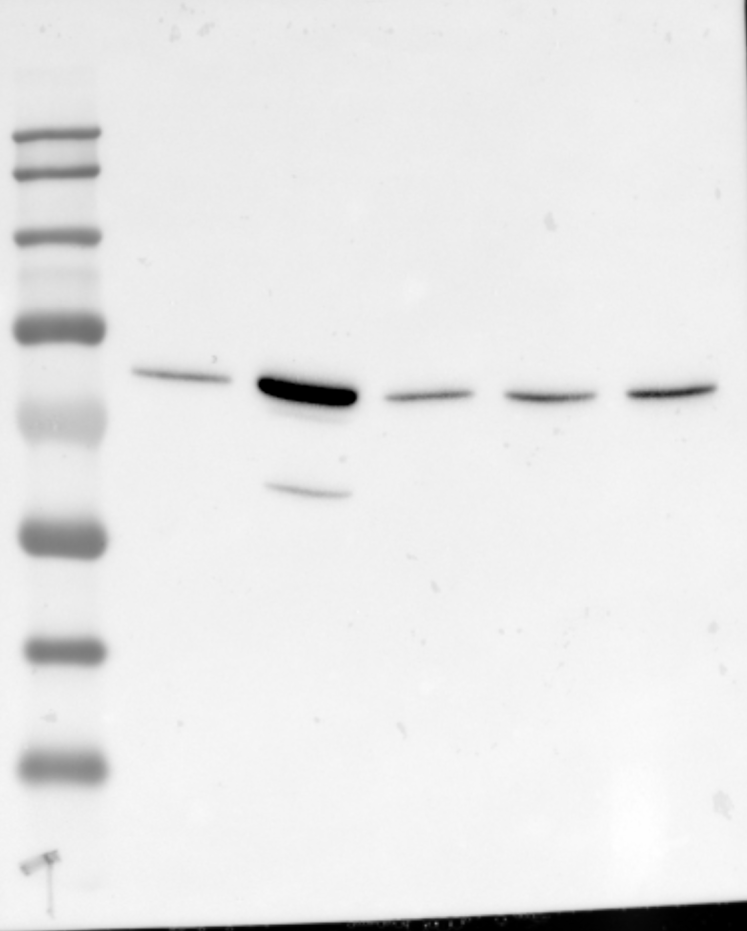

Supplement: Supplementary file 4 — Source data [file 41467_2026_72319_MOESM4_ESM.zip › Source Data/Uncropped immunoblots/Suppl. Fig.4b_TAF1.png]

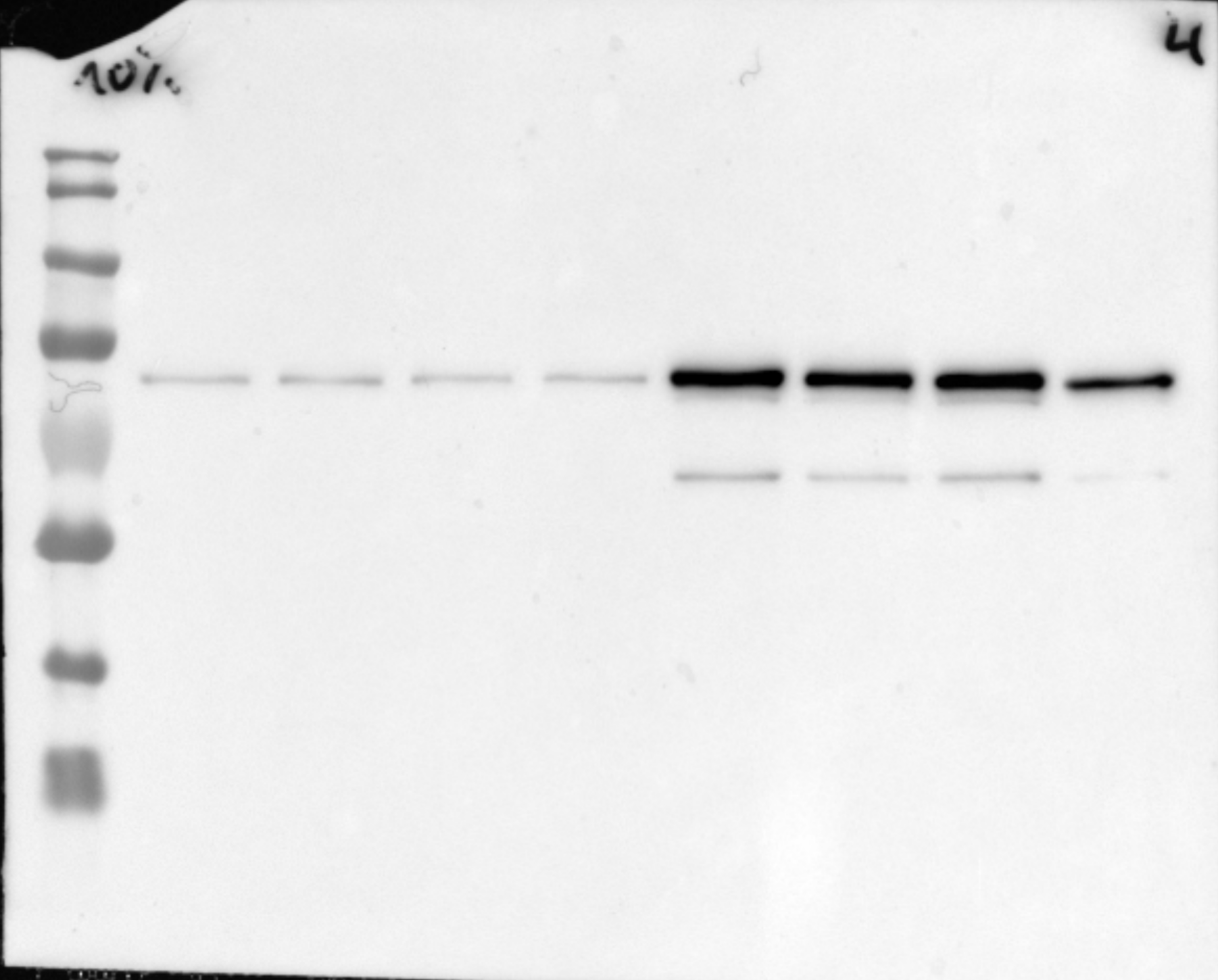

Supplement: Supplementary file 4 — Source data [file 41467_2026_72319_MOESM4_ESM.zip › Source Data/Uncropped immunoblots/Suppl. Fig.4f_GFP.png]

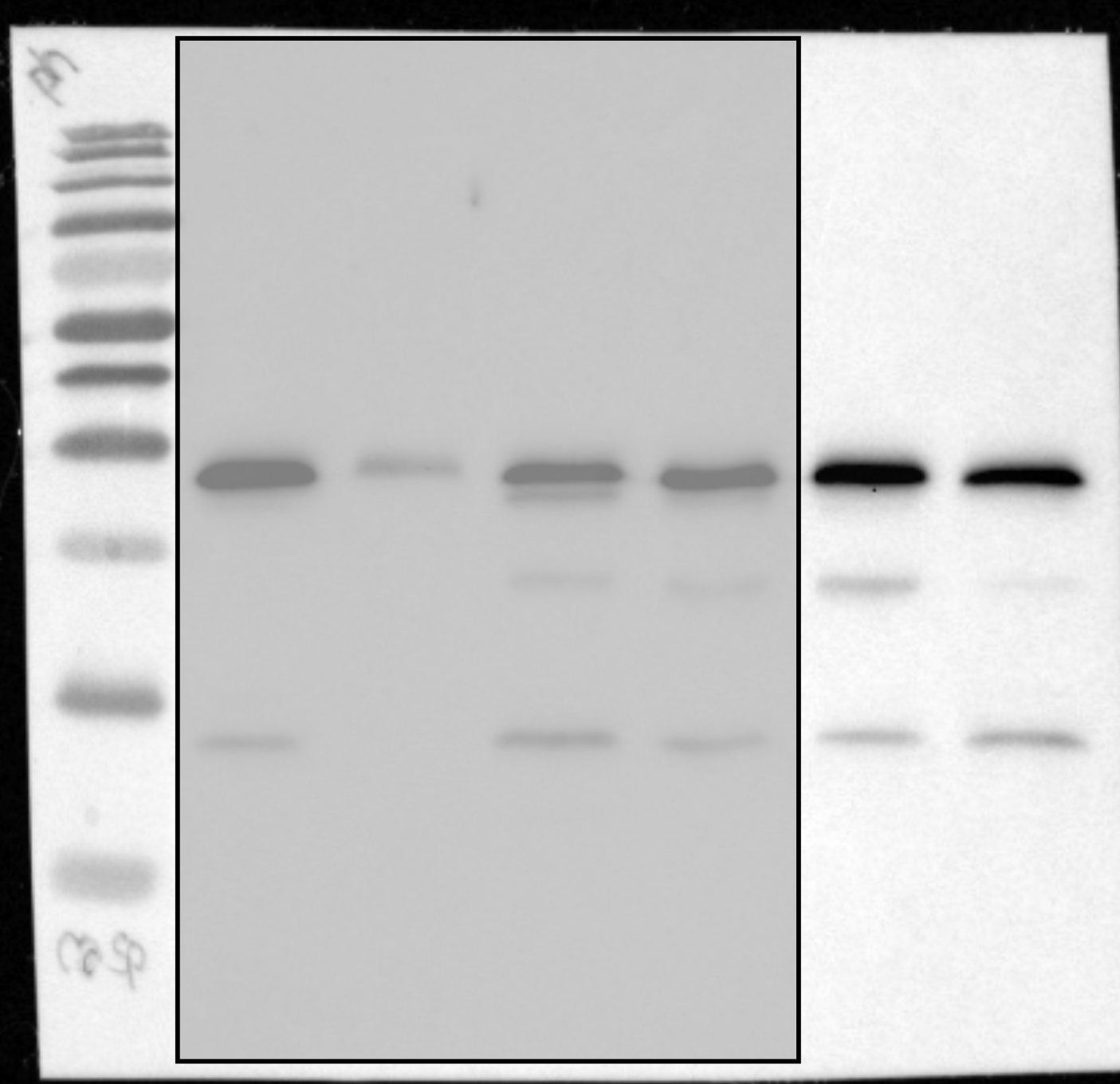

Supplement: Supplementary file 4 — Source data [file 41467_2026_72319_MOESM4_ESM.zip › Source Data/Uncropped immunoblots/Suppl. Fig.6f_Casp3.png]

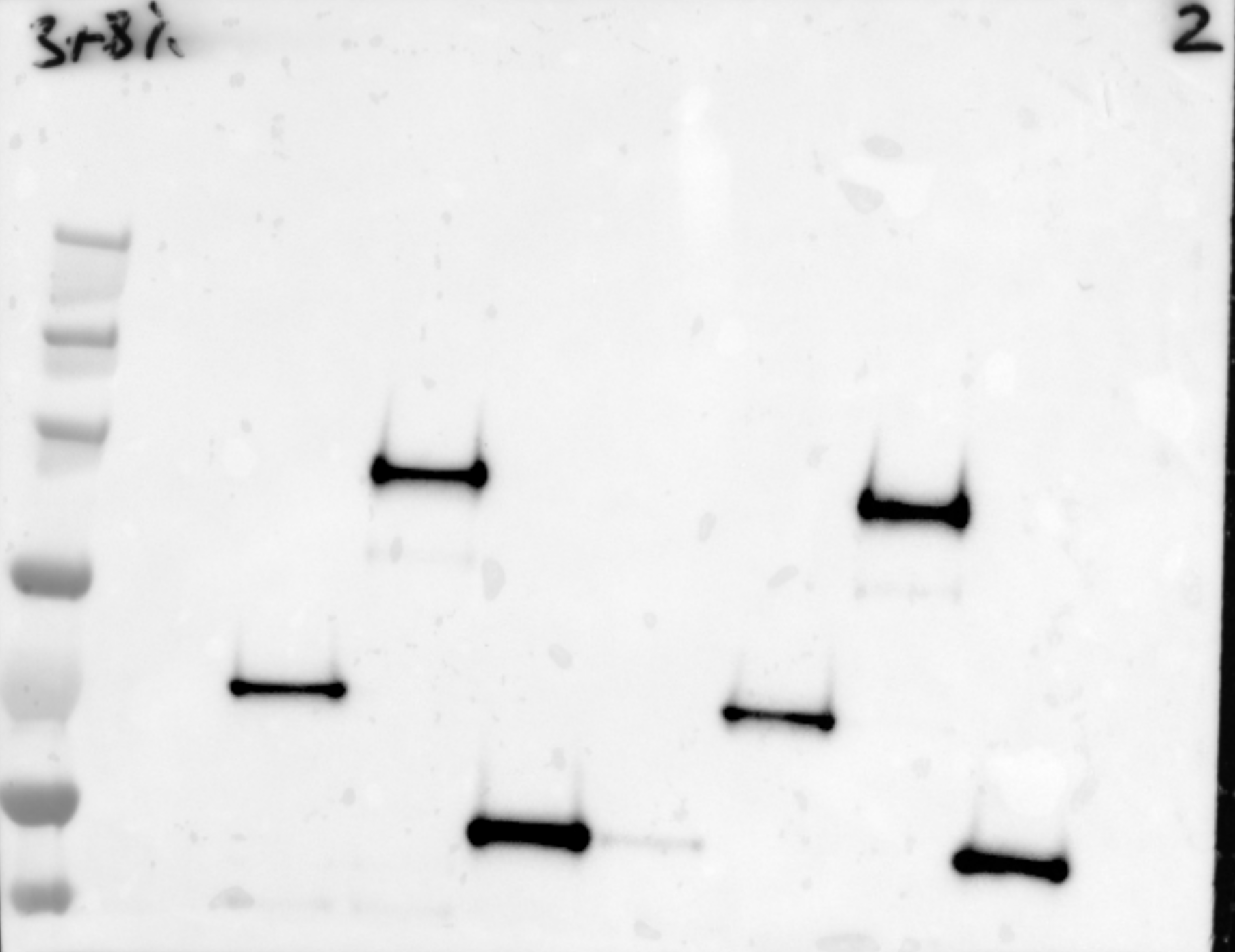

Supplement: Supplementary file 4 — Source data [file 41467_2026_72319_MOESM4_ESM.zip › Source Data/Uncropped immunoblots/Suppl. Fig.4f_HA.png]

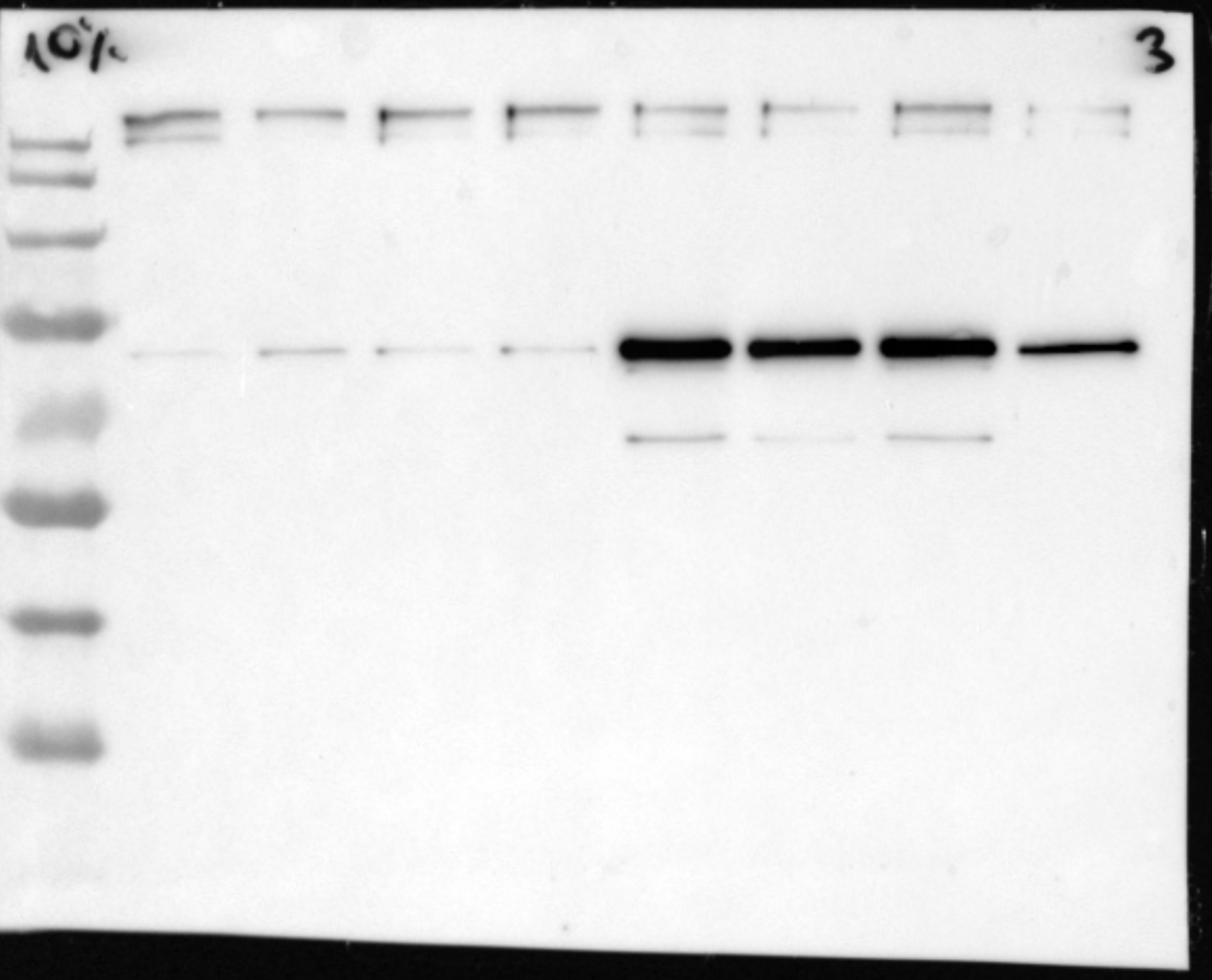

Supplement: Supplementary file 4 — Source data [file 41467_2026_72319_MOESM4_ESM.zip › Source Data/Uncropped immunoblots/Suppl. Fig.4f_TAF1.png]

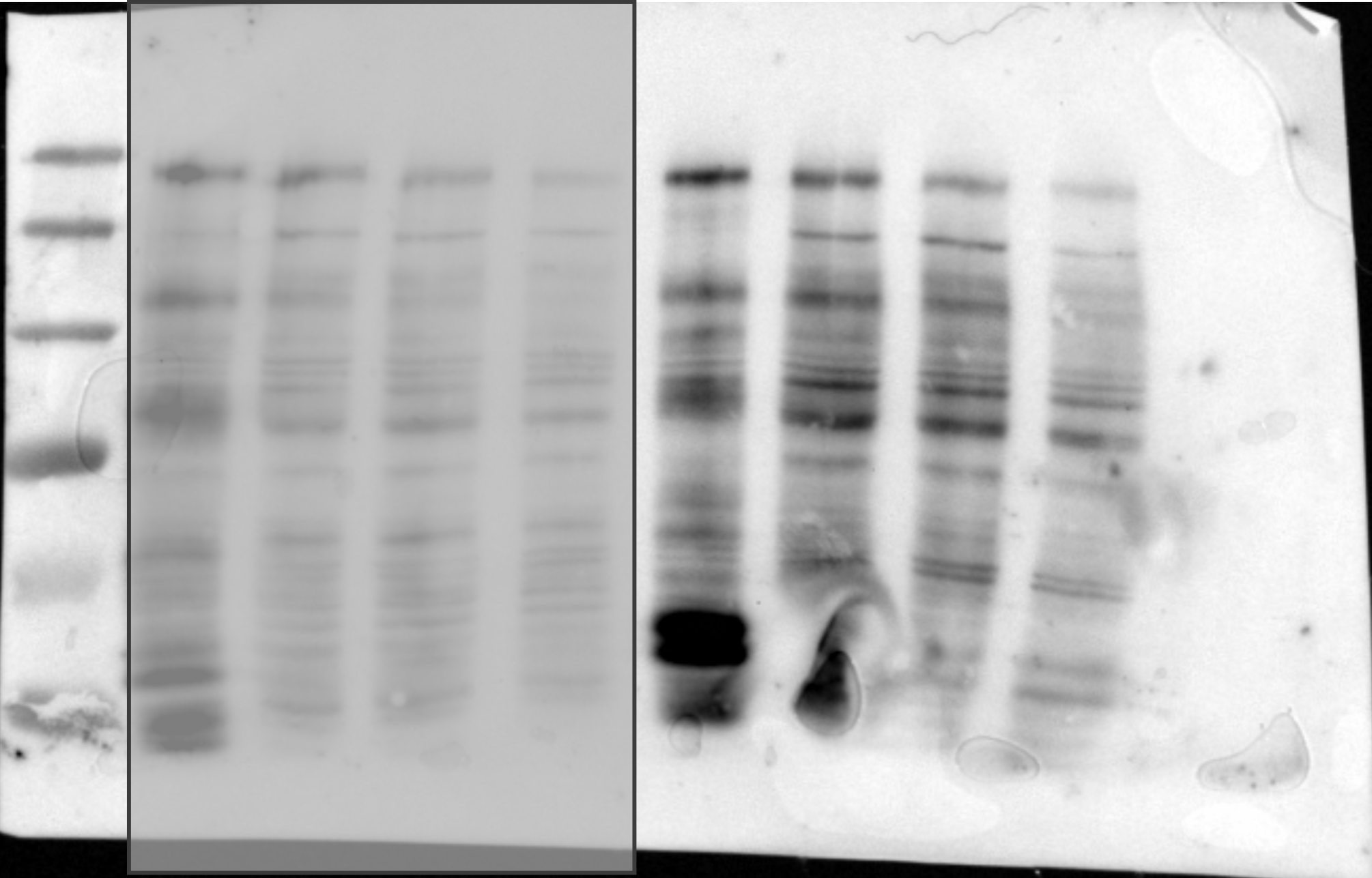

Supplement: Supplementary file 4 — Source data [file 41467_2026_72319_MOESM4_ESM.zip › Source Data/Uncropped immunoblots/Suppl. Fig.6g_BRD4.png]

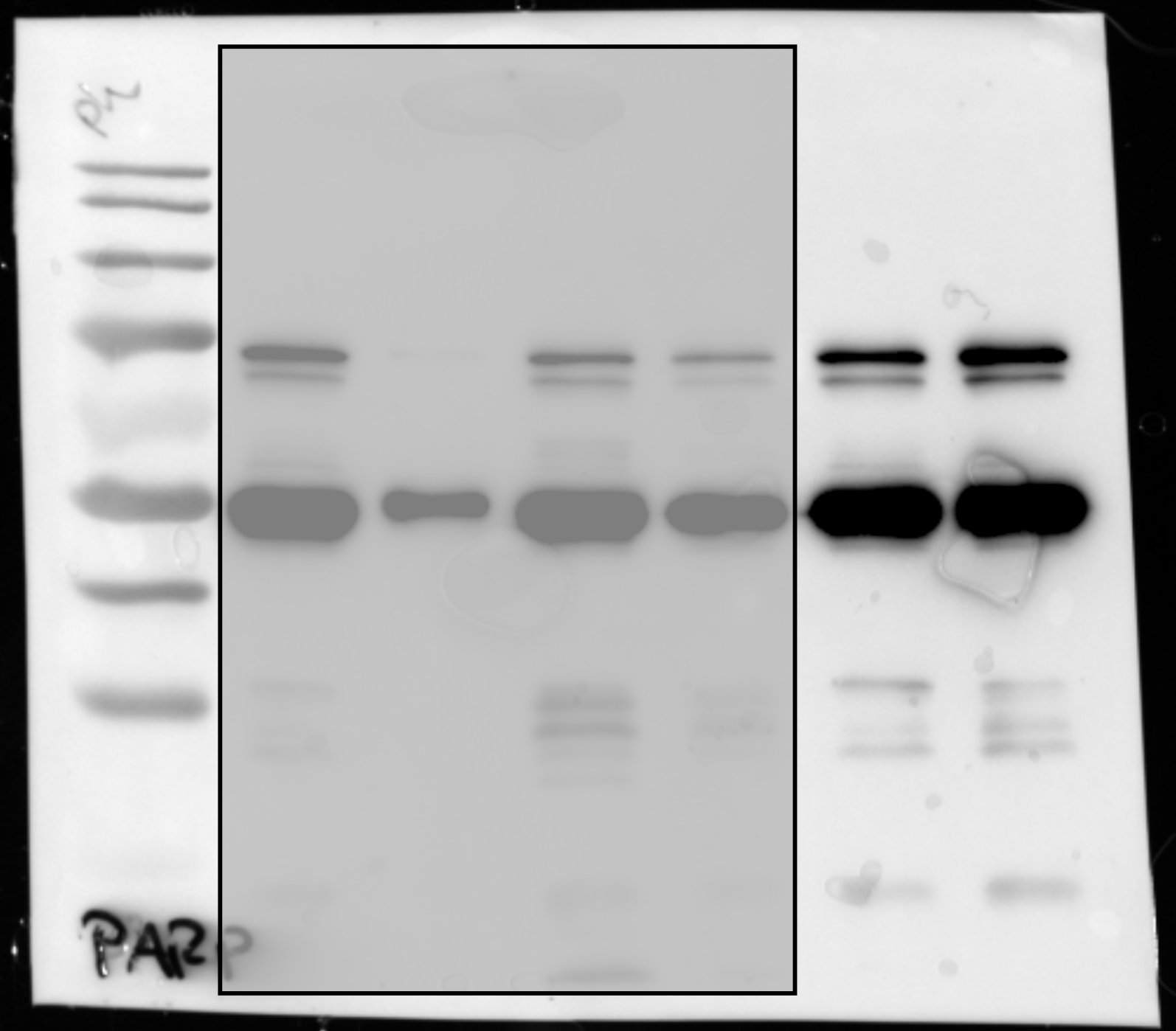

Supplement: Supplementary file 4 — Source data [file 41467_2026_72319_MOESM4_ESM.zip › Source Data/Uncropped immunoblots/Suppl. Fig.6f_cl-PARP.png]

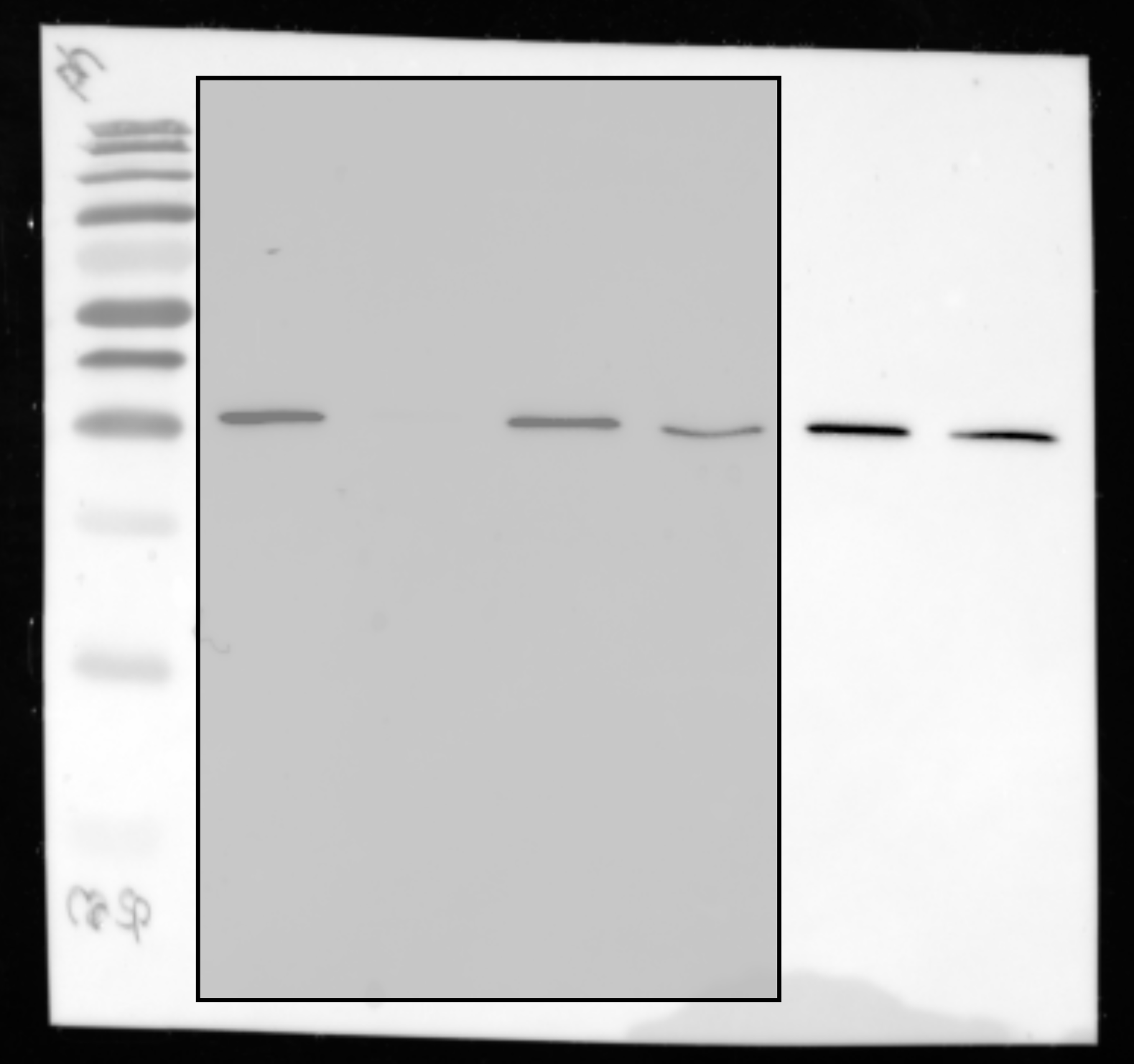

Supplement: Supplementary file 4 — Source data [file 41467_2026_72319_MOESM4_ESM.zip › Source Data/Uncropped immunoblots/Suppl. Fig.6f_GAPDH.png]

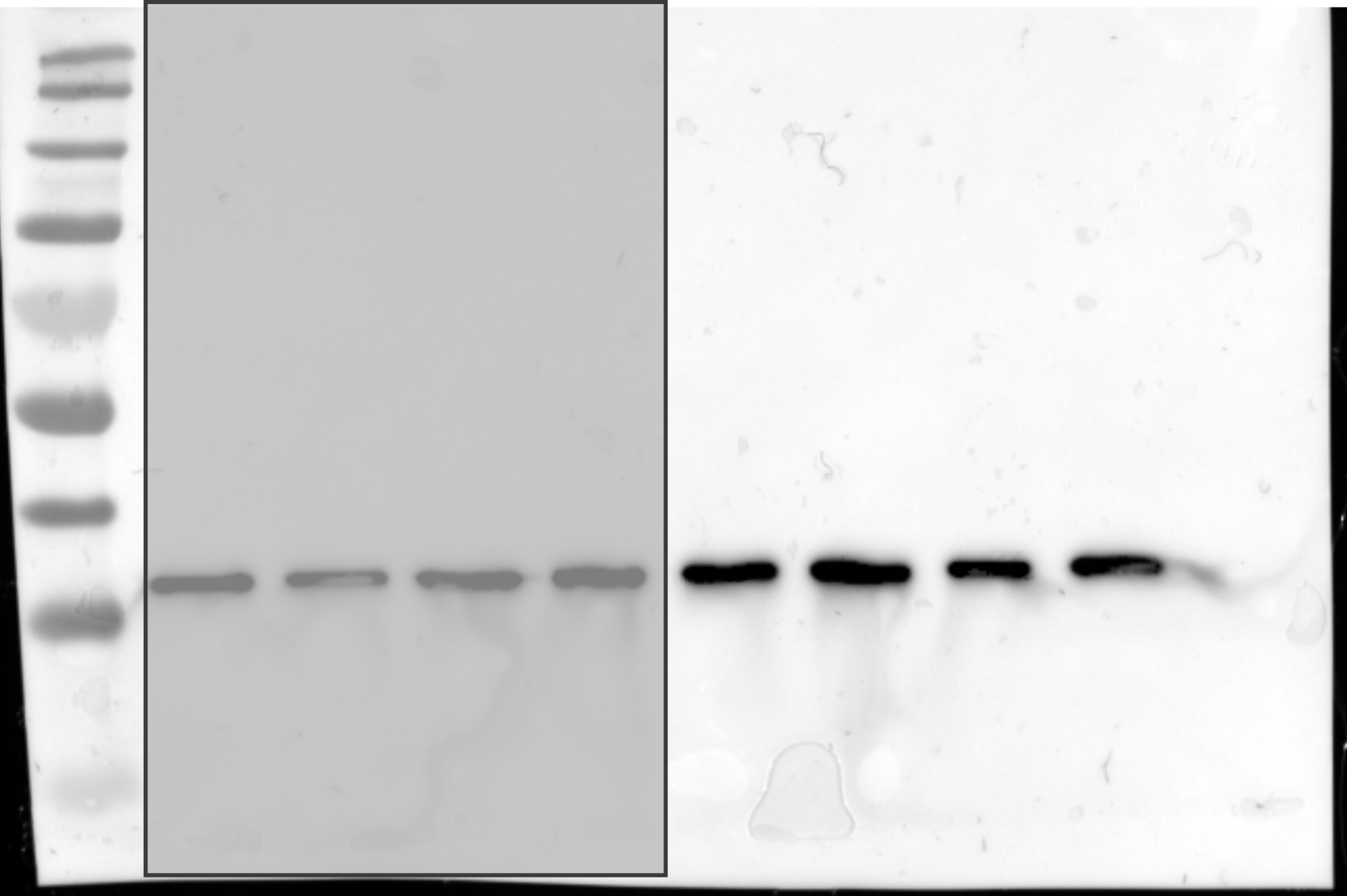

Supplement: Supplementary file 4 — Source data [file 41467_2026_72319_MOESM4_ESM.zip › Source Data/Uncropped immunoblots/Suppl. Fig.6g_GAPDH.png]

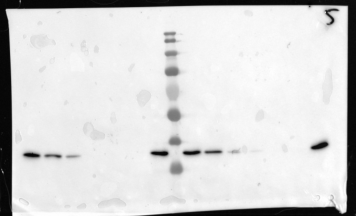

Supplement: Supplementary file 4 — Source data [file 41467_2026_72319_MOESM4_ESM.zip › Source Data/Uncropped immunoblots/Suppl. Fig.5b_CDK9.png]

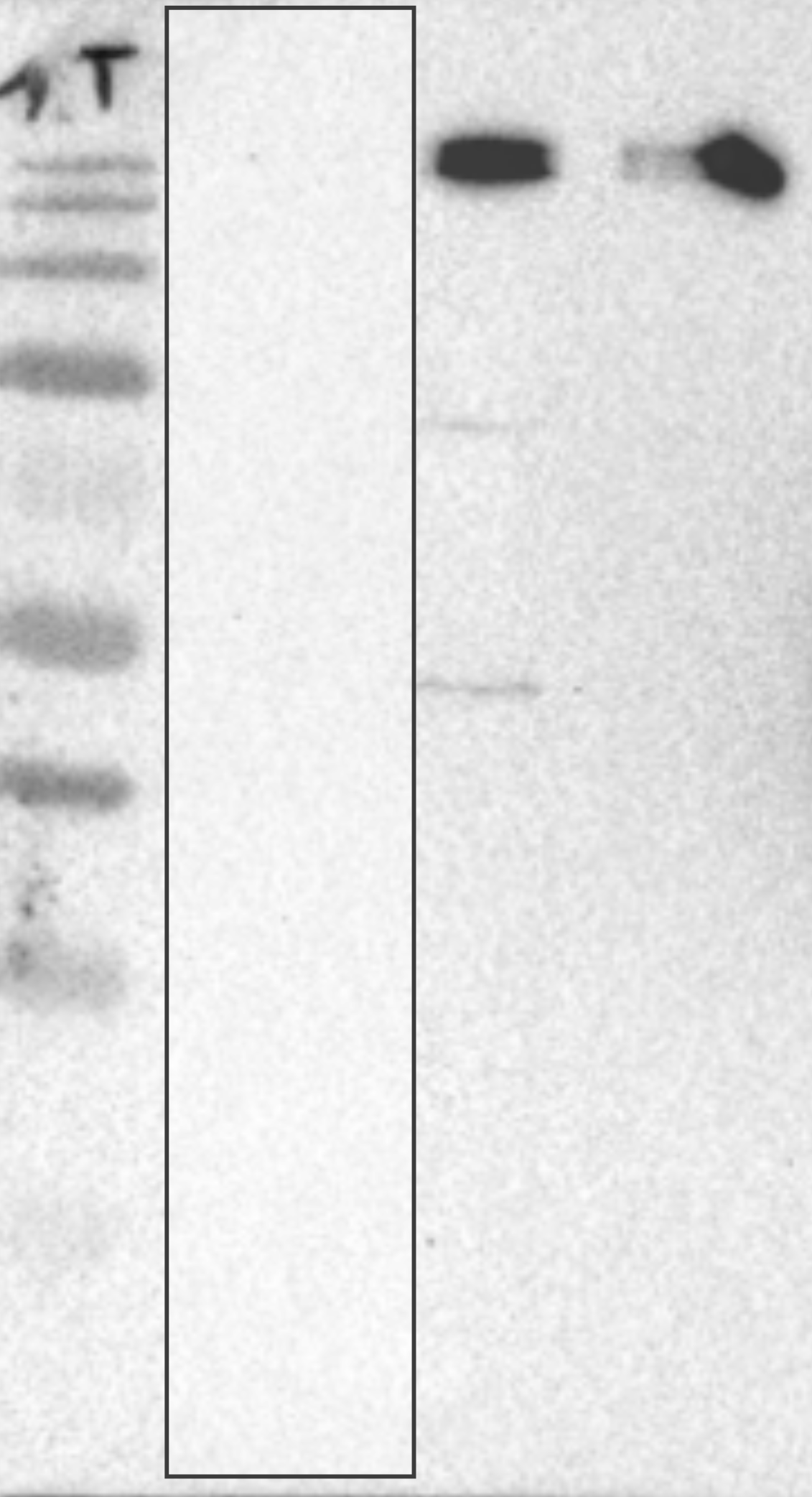

Supplement: Supplementary file 4 — Source data [file 41467_2026_72319_MOESM4_ESM.zip › Source Data/Uncropped immunoblots/Suppl. Fig.1i_TAF1.png]

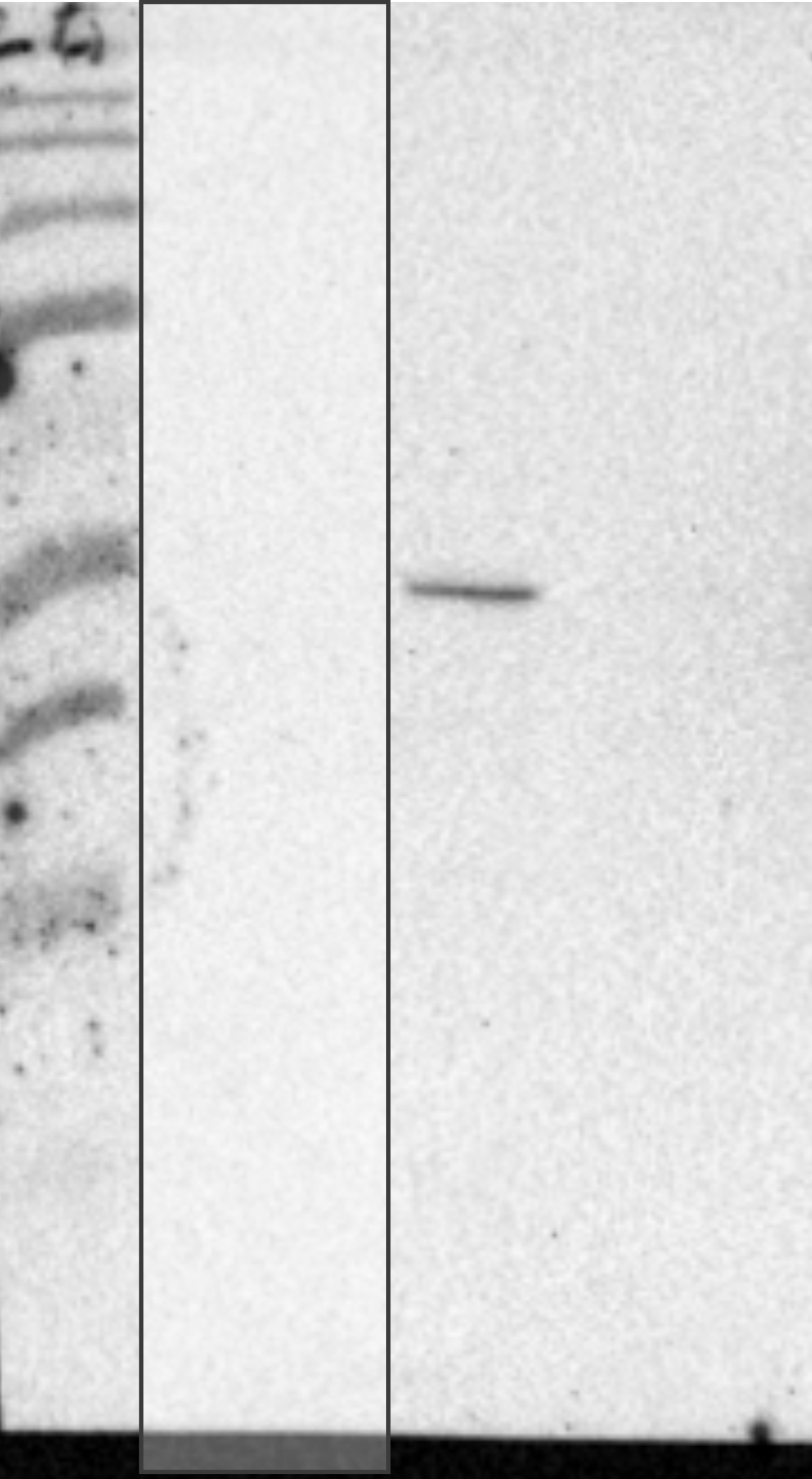

Supplement: Supplementary file 4 — Source data [file 41467_2026_72319_MOESM4_ESM.zip › Source Data/Uncropped immunoblots/Suppl. Fig.1i_GFP.png]

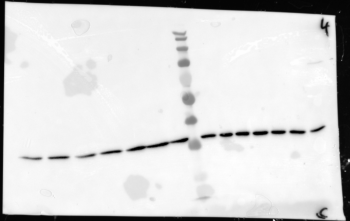

Supplement: Supplementary file 4 — Source data [file 41467_2026_72319_MOESM4_ESM.zip › Source Data/Uncropped immunoblots/Suppl. Fig.5b_GAPDH.png]

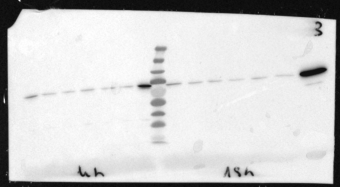

Supplement: Supplementary file 4 — Source data [file 41467_2026_72319_MOESM4_ESM.zip › Source Data/Uncropped immunoblots/Suppl. Fig.5b_GFP.png]

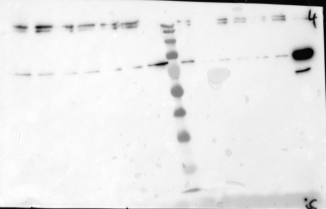

Supplement: Supplementary file 4 — Source data [file 41467_2026_72319_MOESM4_ESM.zip › Source Data/Uncropped immunoblots/Suppl. Fig.5b_TAF1.png]

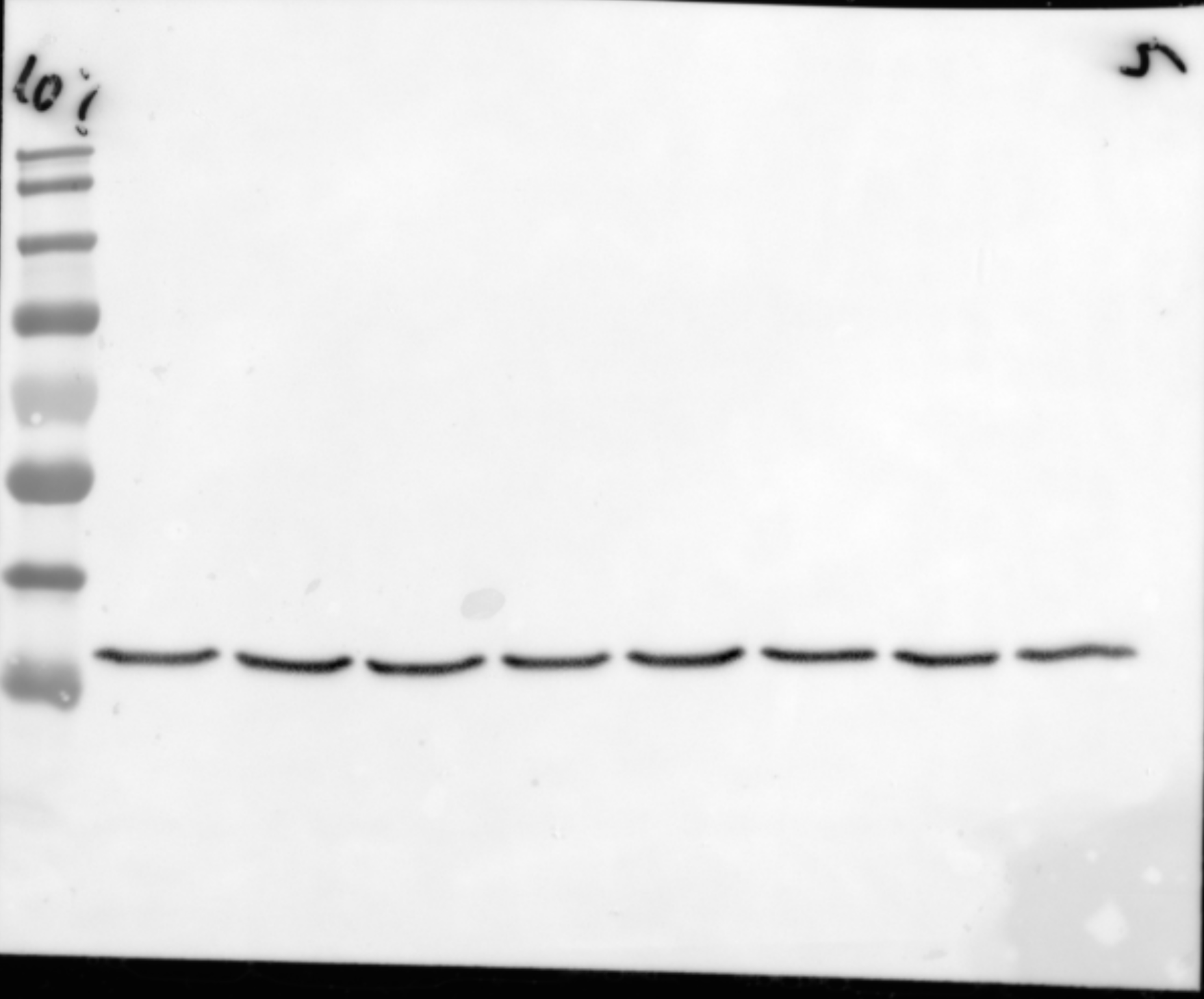

Supplement: Supplementary file 4 — Source data [file 41467_2026_72319_MOESM4_ESM.zip › Source Data/Uncropped immunoblots/Suppl. Fig.4f_GAPDH.png]

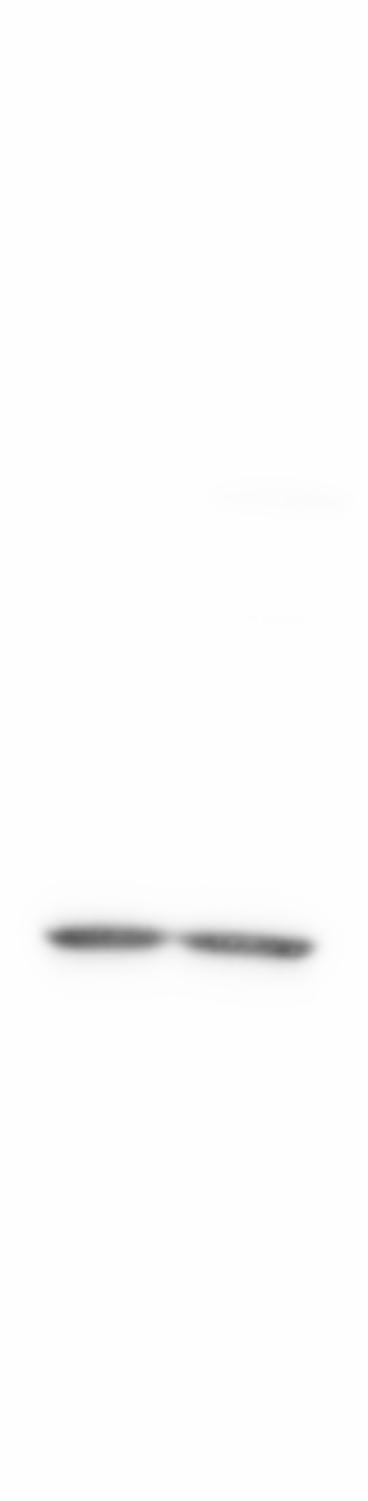

Supplement: Supplementary file 4 — Source data [file 41467_2026_72319_MOESM4_ESM.zip › Source Data/Uncropped immunoblots/Fig.1g_GAPDH.png]
